# Differential pulmonary toxicity and autoantibody formation in genetically distinct mouse strains following combined exposure to silica and diesel exhaust particles

**DOI:** 10.1186/s12989-024-00569-7

**Published:** 2024-02-27

**Authors:** Lisa MF Janssen, Frauke Lemaire, Nora Fopke Marain, Steven Ronsmans, Natasja Heylen, Arno Vanstapel, Greetje Vande Velde, Jeroen AJ Vanoirbeek, Kenneth Michael Pollard, Manosij Ghosh, Peter HM Hoet

**Affiliations:** 1 Environment and Health Unit, KU Leuven, Leuven, Belgium; 2 Department of Molecular Medicine, Scripps Research, La Jolla, CA USA; 3 Laboratory of Respiratory Diseases and Thoracic Surgery (BREATHE), KU Leuven, Leuven, Belgium; 4 Department of Pathology, University Hospitals Leuven, Leuven, Belgium; 5 Department of Imaging and Pathology, Biomedical MRI Unit/MoSAIC, KU Leuven, Leuven, Belgium

**Keywords:** Silica, Silicosis, Diesel exhaust particles, Autoimmunity, Mice, Lung inflammation

## Abstract

**Background:**

Inhalation of airborne particulate matter, such as silica and diesel exhaust particles, poses serious long-term respiratory and systemic health risks. Silica exposure can lead to silicosis and systemic autoimmune diseases, while DEP exposure is linked to asthma and cancer. Combined exposure to silica and DEP, common in mining, may have more severe effects. This study investigates the separate and combined effects of occupational-level silica and ambient-level DEP on lung injury, inflammation, and autoantibody formation in two genetically distinct mouse strains, thereby aiming at understanding the interplay between genetic susceptibility, particulate exposure, and disease outcomes. Silica and diesel exhaust particles were administered to mice via oropharyngeal aspiration. Assessments of lung injury and host response included in vivo lung micro-computed tomography, lung function tests, bronchoalveolar lavage fluid analysis including inflammatory cytokines and antinuclear antibodies, and histopathology with particle colocalization.

**Results:**

The findings highlight the distinct effects of silica and diesel exhaust particles (DEP) on lung injury, inflammation, and autoantibody formation in C57BL/6J and NOD/ShiLtJ mice. Silica exposure elicited a well-established inflammatory response marked by inflammatory infiltrates, release of cytokines, and chemokines, alongside mild fibrosis, indicated by collagen deposition in the lungs of both C57BL/6J and NOD/ShilLtJ mice. Notably, these strains exhibited divergent responses in terms of respiratory function and lung volumes, as assessed through micro-computed tomography. Additionally, silica exposure induced airway hyperreactivity and elevated antinuclear antibody levels in bronchoalveolar lavage fluid, particularly prominent in NOD/ShiLtJ mice. Moreover, antinuclear antibodies correlated with extent of lung inflammation in NOD/ShiLTJ mice. Lung tissue analysis revealed DEP loaded macrophages and co-localization of silica and DEP particles. However, aside from contributing to airway hyperreactivity specifically in NOD/ShiLtJ mice, the ambient-level DEP did not significantly amplify the effects induced by silica. There was no evidence of synergistic or additive interaction between these specific doses of silica and DEP in inducing lung damage or inflammation in either of the mouse strains.

**Conclusion:**

Mouse strain variations exerted a substantial influence on the development of silica induced lung alterations. Furthermore, the additional impact of ambient-level DEP on these silica-induced effects was minimal.

**Supplementary Information:**

The online version contains supplementary material available at 10.1186/s12989-024-00569-7.

## Background

Airborne particulate matter (PM) inhalation poses a significant threat to long-term respiratory health with adverse effects such as interstitial lung diseases, increased susceptibility to infections, and chronic obstructive pulmonary disease. Certain occupations present a heightened risk to workers due to exposure to various airborne particulates. One well-known airborne particulate is crystalline silica, a naturally occurring mineral commonly found in rocks, sand, and soil, presenting a major occupational inhalation hazard for workers in various industries, including construction and mining [1]. Epidemiological data from regulatory agencies including the Occupational Safety and Health Administration (OSHA) in the U.S [2]. and EU-OSHA in Europe, have estimated that over 2 million workers in the U.S. and approximately 5.3 million workers in Europe are potentially exposed to hazardous levels of silica dust.

Silicosis, a chronic lung disease characterized by inflammation and nodular fibrosis, is a well-known health issue stemming from silica dust inhalation. Although silicosis is not a new disease, recent outbreaks occurred in young workers involved in jeans sandblasting and in workers handling artificial granite or engineered stone [3, 4], showing that silica dust exposure and silicosis remain relevant to this day. Beyond silicosis, inhalation of silica has also been linked to systemic autoimmune diseases (SAD) such as systemic lupus erythematosus (SLE), systemic sclerosis (SSc), and rheumatoid arthritis (RA) [5, 6]. This association underscores the connection between inhalation exposures and systemic effects, raising further concerns about the broader health implications for exposed workers.

In the context of silicosis and SAD associated with inhalation of silica particles, the question arises whether there is an association between both disease pathways. Cases of autoimmune diseases associated with prior silica exposure have been documented independently of a silicosis diagnosis [7]. Additionally, silicosis cases have been observed where specific autoantibodies are significantly present [1]. Doll et al. highlighted that silicosis patients exhibited an increased prevalence of particular autoantibodies [8]. However, because the presence of these autoantibodies has not been correlated with pulmonary alterations in silicosis, the role of these autoantibodies in the pathophysiology of silicosis remains unclear. Additionally, research conducted by Mayeux et al. [9] in a murine model exposed to silica, demonstrated a close association between silicosis, markers of lung inflammation and fibrosis, lung biomarkers, and autoantibodies against extractable nuclear antigens. Given the shared inflammatory pathways in the initial stages of silicosis development and the presumed pathogenesis of silica-associated autoimmunity, an intricate interplay between these disease states is not unthinkable. Nevertheless, investigating this intricate relationship is a complex task, and the development of an animal model that more accurately resembles human silicosis holds promise for yielding novel insights.

Another airborne particulate common in mining and other dusty trades is diesel exhaust particles (DEP). DEP are present in diesel engine emission, which is a highly complex mixture of chemical substances in either gas or particle form. Exposure has been associated with enhanced allergic sensitization, development and aggravation of asthma, chronic bronchitis, decreased lung function, airway inflammation, decreased vascular function and development of cancers, as reported in epidemiological studies [10–15]. Similarly, crystalline silica has been classified as a class 1 carcinogen by International Agency for Research on Cancer (IARC), based on sufficient evidence for carcinogenicity in both humans and experimental animals [16]. However, the IARC working group also stated that the carcinogenicity was not found in all industrial circumstances, and their conclusion remained somewhat controversial. The risk tends to be more pronounced with pre-existing silicosis and in the mining industry [17]. However, the current study focuses on the immediate respiratory and immunological effects of silica and DEP, acknowledging their known carcinogenic potential but emphasizing non-cancerous outcomes due to the scope and duration of our investigation.

While extensive research exists on the individual health effects of DEP and silica exposure, little is known about the impact of their combined exposure. Combined exposure to silica and DEP is common during mining operations, including hydraulic fracturing for oil or gas, as well as above- and underground mining operations [18–21]. Studies suggest that combined exposure to different types of particles or other environmental factors, such as viruses, may induce more pronounced effects compared to those caused by the individual compounds [22, 23]. Only one study [23] investigated the combined exposure of silica and DEP particles, using a high dose of DEP representing occupational exposure, looking at inflammatory outcomes and susceptibility to infection, as opposed to the current study, in which a lower dose of DEP, representing ambient DEP exposure was used. Moreover, the current study includes, apart from inflammatory endpoints, also functional end points such as lung function.

Research has shown the substantial influence of genetic susceptibility on the extent of silicosis or pulmonary inflammation elicited by specific triggers [24–26]. Furthermore, genetic predisposition assumes even greater significance in the context of systemic autoimmune diseases [27]. As genetic susceptibility plays a pivotal role not only in the development of pulmonary inflammation but also in the broader spectrum of autoimmunity, our investigation incorporated two murine strains. To provide comprehensive insights, we selected the extensively characterized C57BL/6J strain, well-studied in both silicosis and autoimmunity, and the NOD/ShiLtJ strain, distinguished by its chronic inflammatory phenotype and heightened proclivity for autoimmune responses. Specifically, we investigated how exposure to DEP, silica particles, and their combination impacts lung inflammation, lung function, airway hyperreactivity and local and systemic antinuclear antibodies in two mouse strains with differences in sensitivity.

## Results

### C57BL/6J and NOD/ShiLtJ mice display differences in lung volumes and baseline lung function in response to silica and DEP exposure

**Fig. 1:**
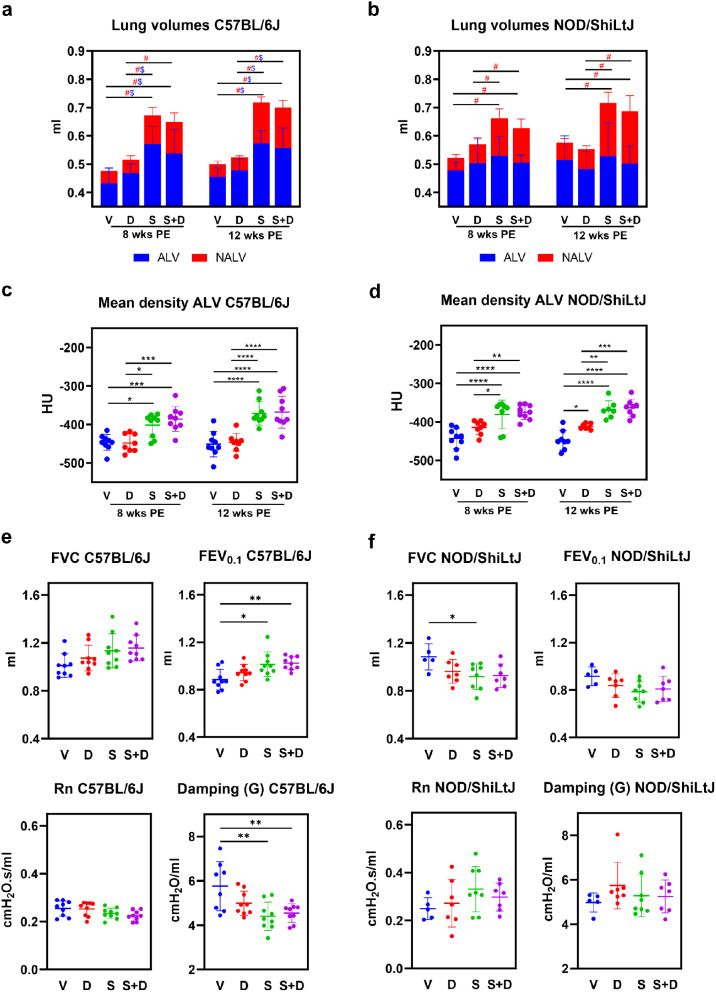
(a) Stacked column plots representing non-aerated lung volumes (NALV) (ml) and aerated lung volumes (ALV) (ml) [resulting in total lung volumes (TLV) (ml)] in C57BL/6J and NOD/ShiLtJ mice. Data are presented as mean ± SD. Experimental groups were compared using repeated measures Two-Way ANOVA or mixed model in case of missing values with Tukey correction for multiple testing. Significant differences are represented as follows: $*p* < 0.05 between ALV, # *p* < 0.05 between NALV. *N* = 7–9 mice/group. (b) Mean aerated lung volume (HU) in DEP, silica, silica + DEP and vehicle exposed C57BL/6 and NOD/ShiLtJ mice. Data are presented as individual values with mean ± SD. *p < 0.05, **p < 0.01, ***p < 0.001 and ****p < 0.0001 by repeated measures Two-way ANOVA or mixed model in case of missing values with Tukey correction. *N* = 7–9 mice/group. (c) Forced vital capacity (FVC), Forced Expiratory Capacity in 0.1 s (FEV_0.1_) and Tissue Damping (G) in DEP, silica, silica + DEP and vehicle exposed C57BL/6 and NOD/ShiLtJ mice. Data are represented as individual values with mean ± SD. *p < 0.05, **p < 0.01, ***p < 0.001 and ****p < 0.0001 by One-way ANOVA with Tukey correction. *N* = 5–9 mice/group

In vivo micro-computed tomography (micro-CT, µCT) scans were performed to evaluate aerated (ALV) and non-aerated lung volumes (NALV) (ml), total lung volumes (ml) (TLV), mean total lung density (Hounsfield units [HU]), mean aerated lung density (HU), and mean non-aerated lung density (HU). Scans were performed at two different time points, 8 and 12 weeks after the start of the experiment (see “Methods” Fig. 9 for experimental design). Visual inspection of transverse sections from the micro-CT images revealed a visibly higher number of dense areas in silica and silica + DEP exposed, but not DEP exposed mice compared to vehicle mice (Additional Fig. 1). When determining the aerated and non-aerated lung volumes (based on delineated area of interest and a cut-off in density), non-aerated lung volumes (NALV), which directly quantifies inflammatory and fibrotic disease burden [28], were higher in both silica exposed C57BL/6J (Fig. 1a) and NOD/ShiLtJ (Fig. 1b) mice compared to vehicle and DEP exposed mice, both in week 8 and week 12. Responses were in a similar extent, as fold changes over vehicle were not significantly different between strains (Additional File 1). These results were reflected in the mean density of the scans, as it was observed that silica exposed mice (both strains) demonstrated significantly higher mean lung densities than DEP and vehicle mice (Additional Fig. 3), primarily due to higher mean aerated lung densities in both strains (Fig. 1c&d). Moreover, DEP exposed NOD/ShiLtJ mice exhibited a higher mean aerated lung density compared to vehicle mice, but only at 12 weeks post-exposure (Fig. 1d). The density of aerated and non-aerated lung volumes reflects the composition of the alveoli and the surrounding tissues, including the epithelial layer, capillaries, extracellular matrix, and small airways, respectively. Higher density is typically associated with lung edema and the accumulation of inflammatory cells.

Remarkably, also total lung volumes (TLV) were significantly higher in silica exposed mice compared to vehicle exposed mice, in both mouse strains (Fig. 1a&b), and silica exposed C57BL/6J mice also exhibited higher aerated lung volumes (ALV) compared to vehicle and DEP exposed mice (Fig. 1a). These results can be attributed to a compensatory mechanism known to happen in mice during fibrosis or inflammation, but not in humans [28]. However, in NOD/ShiLtJ mice (Fig. 1b), no differences in ALV could be observed between experimental groups. The differences in response between the strains for ALV is confirmed by the fold change comparisons, which were significantly different for silica and silica + DEP (Additional File 1). Additionally, TLV and NALV, but not ALV, were significantly higher in vehicle exposed NOD/ShiLtJ mice compared to C57BL/6J mice (Additional Fig. 2). These findings suggest that NOD/ShiLtJ mice have a higher baseline inflammatory state compared to C57BL/6J mice. DEP exposed mice did not show any significant differences in their aerated, non-aerated, or total lung volumes compared to vehicle exposed mice, and none of the effects induced by silica were significantly enhanced by DEP co-exposure.

These results were further reflected in the baseline lung function tests. In silica and silica + DEP exposed C57BL/6J mice, inspiratory capacity (IC) (Additional Fig. 4) and forced expiratory volume in the first 0.1 s (FEV_0.1_) (Fig. 1e) were significantly higher compared to vehicle mice (Fig. 1c). In contrast, these lung function biomarkers did not show significant increases upon silica and/or DEP exposure in NOD/ShiLtJ mice (Fig. 1f). On the contrary, FVC was observed to be lower in silica exposed NOD/ShiLtJ mice compared to vehicle mice. Additionally, tissue damping (G) (Fig. 1f) and tissue elastance (H) (Additional Fig. 4), but not tissue hysteresivity (G/H) (Additional Fig. 4), were significantly lower in silica and silica + DEP exposed C57BL/5J mice, but not in NOD/ShiLtJ mice. DEP exposed mice did not show significant differences from vehicle mice for the included parameters measured by FlexiVent, and DEP co-exposure did not significantly enhance the effects induced by silica exposure. No significant differences were observed between groups for Newtonian airway resistance (Rn) (Fig. 1e,f) and peak expiratory flow (PEF) (Additional Fig. 4). Differences in responses between the strains were also statistically confirmed by fold change comparisons, as outlined in Additional File 1.

### Silica and DEP exposure elicit differential airway hyperreactivity responses in C57BL/6J and NOD/ShiLtJ mice

**Fig. 2:**
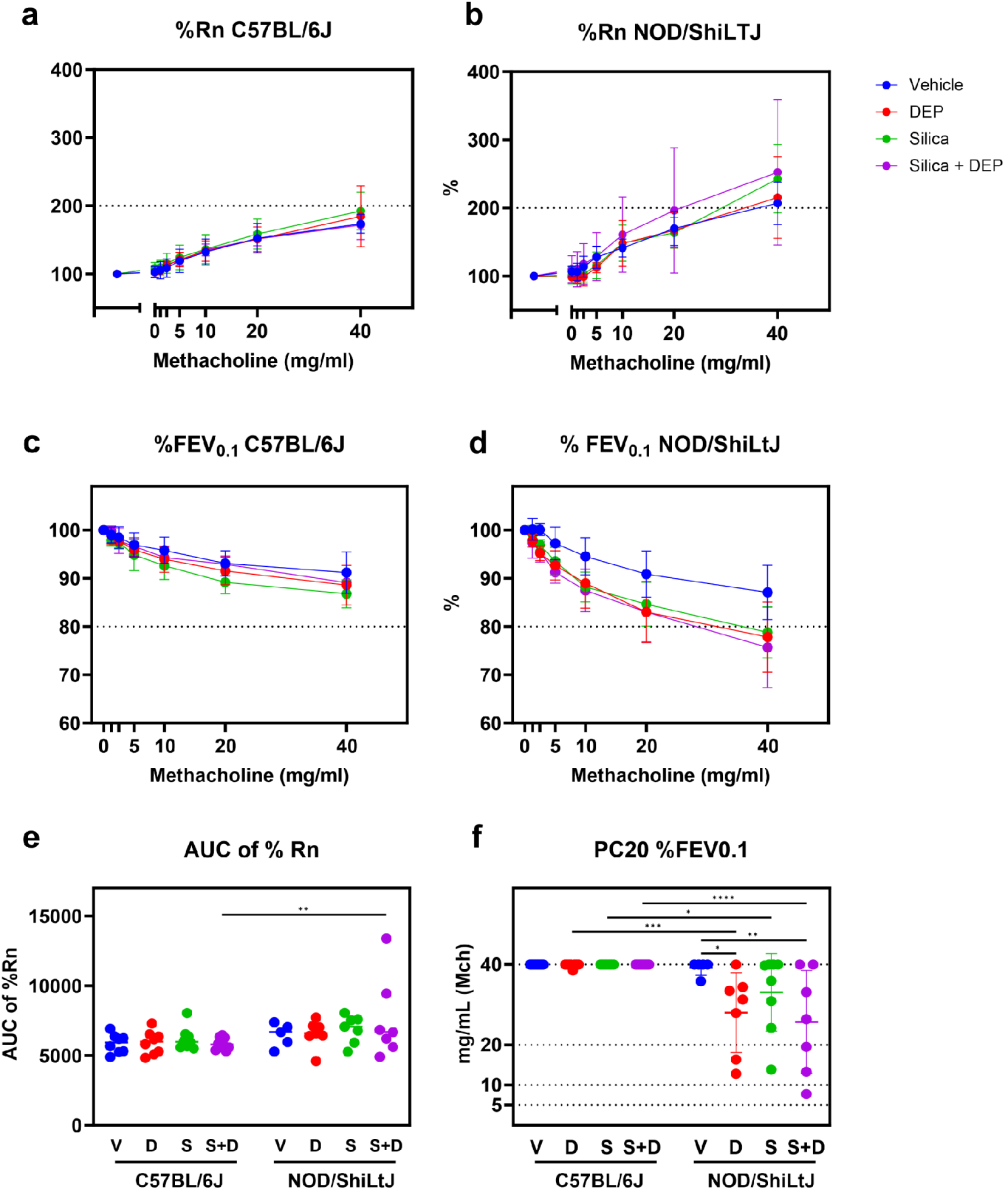
Newtonian airway resistance (Rn) (% of baseline) (a&b) and FEV_0.1_ (% of baseline) (c&d) in response to increasing methacholine challenge in DEP, silica, silica + DEP and vehicle exposed C57BL/6 and NOD/ShiLtJ mice (e) Area under the curve of %Rn. (f) PC20 of %FEV_0.1_. Data are represented as mean ± SD. *p < 0.05, **p < 0.01, ***p < 0.001, ****p < 0.0001 with Two-way ANOVA with Tukey corrections for multiple testing

FEV_0.1_ and airway resistance (Rn), both represented as % of baseline, were measured at baseline and after methacholine challenge (0, 1.25, 2.5, 5, 10, 20 and 40 mg/ml) to assess airway hyperreactivity (AHR) (Fig. 2). None of the experimental groups of C57BL/6J mice reached cut-off values for hyperreactivity as assessed by %FEV_0.1_ (Fig. 2c) and %Rn (Fig. 2a). DEP, silica and silica + DEP exposed NOD/ShiLtJ mice, on the other hand, showed a significantly enhanced decrease in %FEV_0.1_ upon methacholine challenge (Fig. 2d), reaching a 20% decrease with 20–40 mg/ml methacholine (Fig. 2f). %Rn of baseline did not show significant differences between experimental groups for NOD/ShiLtJ mice (Fig. 2b), but all the experimental group means reached the cut-off value of 200%. In conclusion, NOD/ShiLtJ mice seem to display hyperreactivity, which is more pronounced with exposure to DEP and/or silica, while this response is lacking in C57BL/6J mice.

### NOD/ShiLtJ mice show higher extent of lung inflammatory response upon silica exposure, based on lung histology and bronchoalveolar lavage fluid cell counts

**Fig. 3:**
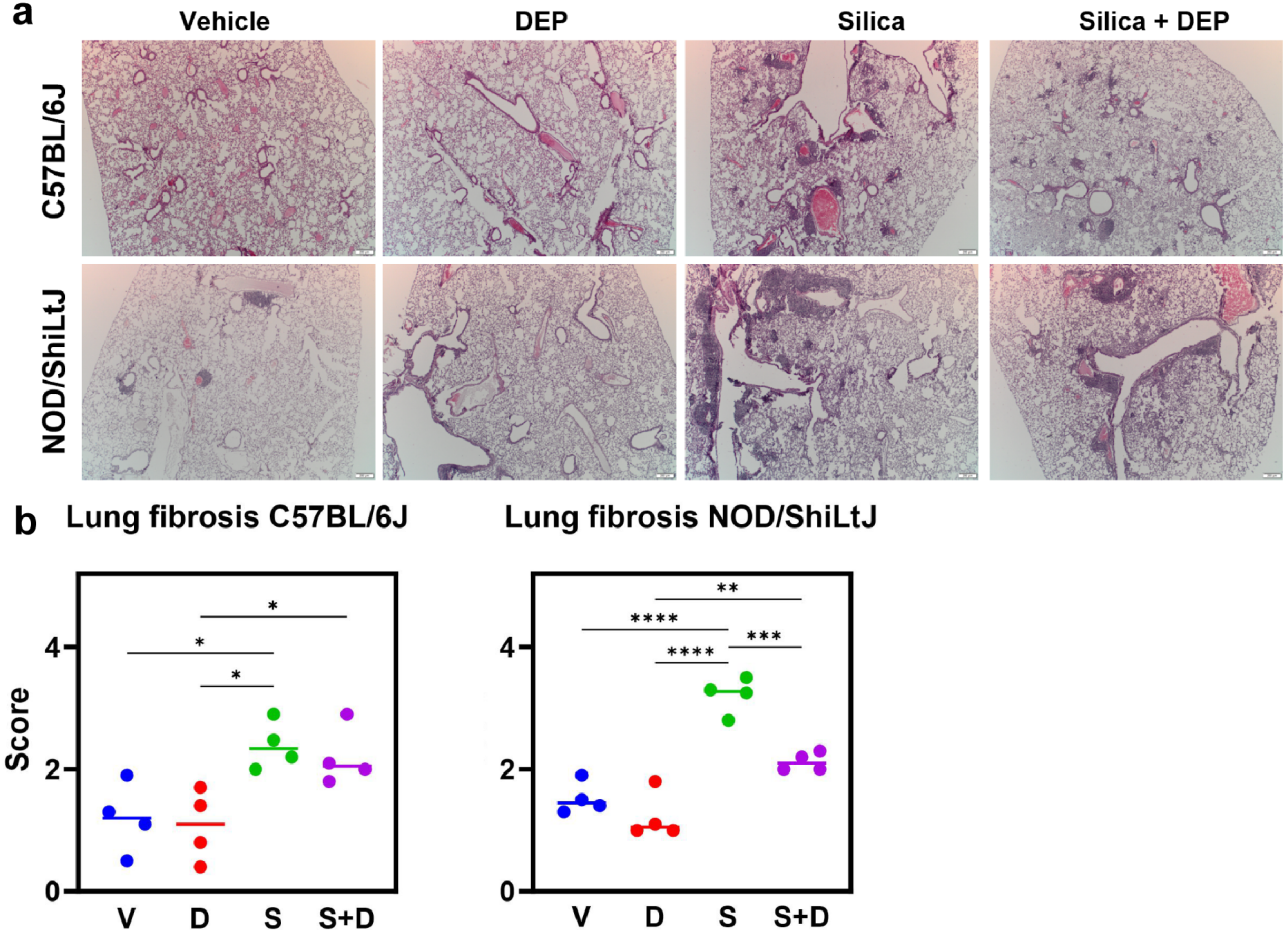
(a) Representative sections of H&E-stained lung tissue slides (5 μm) of vehicle-, DEP-, silica- and silica + DEP exposed C57BL/6J and NOD/ShiLtJ mice. (b) Pulmonary fibrosis scores of vehicle, DEP, silica and silica + DEP exposed C57BL/6J and NOD/ShiLtJ mice using the modified Ashcroft grading scale [29]. Data are represented as mean ± SD. *p < 0.05, **p < 0.01, ***p < 0.001, ****p < 0.0001 with One-way ANOVA. *n* = 4 mice/group

The micro-CT and lung function analyses were complemented by qualitative histological examination of lung tissue, and analysis of inflammatory markers in bronchoalveolar lavage (BAL) fluid (Additional File 2). H&E-stained lung sections showed mononuclear inflammatory infiltrates around bronchioles and vasculature as well as interstitially (Fig. 3a) after silica and silica + DEP exposure in both strains. In silica-only exposed C57BL/6J mice, infiltrates were more apparent than in the silica + DEP exposed C57BL/6J mice. In NOD/ShiLtJ mice, similar presentations of inflammatory infiltrates were observed in silica and silica + DEP exposed mice. In addition, also vehicle and DEP exposed NOD/ShiLtJ mice presented with several inflammatory infiltrates, but in a lesser extent compared to the silica and silica + DEP group. Furthermore, silica and silica + DEP exposed mice also demonstrated presence of bi- and multinucleated cells, indicative of presence of giant cells, a feature of a chronic inflammatory state in the lungs.

Notably, NOD/ShiLtJ mice showed more abundant inflammatory infiltrates upon silica exposure compared to C57BL/6J mice. The inflammatory infiltrates in the vehicle groups of NOD/ShiLtJ mice, together with results from the exposed groups, confirms a predisposition to an inflammatory phenotype. This observation aligns with the higher non-aerated lung volume observed in vehicle exposed NOD/ShiLtJ mice compared to C57BL/6J mice (Additional Fig. 2).

**Fig. 4:**
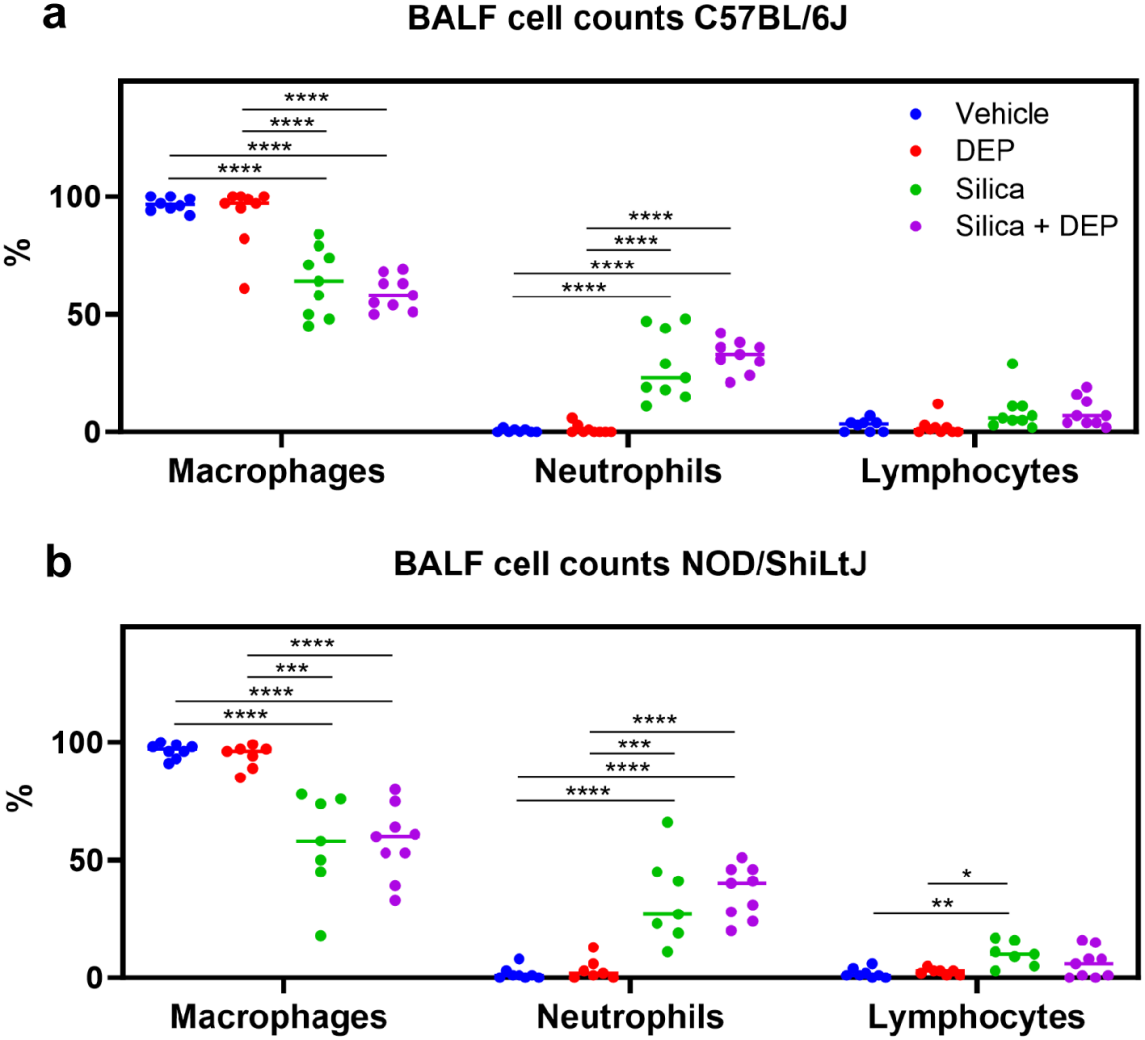
(a) % differential BAL fluid cell counts in C57Bl/6J mice, and (b) NOD/ShiLtJ mice. Data are represented as mean ± SD. *p < 0.05, **p < 0.01, ***p < 0.001, ****p < 0.0001 with One-way ANOVA and Tukey correction for multiple testing. *n* = 7–9/group

Micro-CT scans and lung function measures alone do not allow for the differentiation between fibrosis and lung inflammation. Therefore, we conducted an additional specific evaluation of lung fibrosis using H&E and Sirius red-stained lung tissue. Sirius Red stained lung tissue showed areas with collagen deposition in silica and silica + DEP exposed mice in both strains (Additional Fig. 5). In addition, a standardized grading scale was used to quantify the degree of pulmonary fibrosis in the H&E-stained lung sections. Individual and average values of pulmonary fibrosis scores for each experimental group are shown in Fig. 4b. Silica and silica + DEP exposed C57BL/6J mice were scored significantly higher than vehicle and DEP exposed mice. Silica, but not silica + DEP exposed NOD/ShiLtJ mice were scored significantly higher than vehicle and DEP exposed mice. However, although mild fibrosis was observed as shown by the fibrosis scores and the collagen deposition, none of the sections showed overt fibrosis, as none of the scores were higher than four on a scale of eight.

Silica and silica + DEP exposed mice (both strains) showed a relative increase of neutrophils, resulting in a relative decrease in macrophages (Fig. 4). Fold change comparisons showed how this increase in neutrophils was significantly more apparent in NOD/ShiLtJ mice compared to C57BL/6J mice (Additional File 1). When looking at the absolute cell numbers (total counts) (Additional Fig. 6), it was evident that the number of macrophages increased upon silica and silica + DEP exposure (C57BL/6J) or remained consistent (NOD/ShilLtJ) across all experimental groups. Eosinophil numbers did not increase upon silica and/or DEP exposure (Additional Fig. 6, absolute counts), while lymphocyte numbers showed a mild increase in numbers, significant in NOD/ShiLtJ mice, but not in C57BL/6J mice.

### BAL fluid inflammatory cytokines show similar responses upon silica and DEP exposure in C57BL/6J and NOD/ShiLtJ mice

**Fig. 5:**
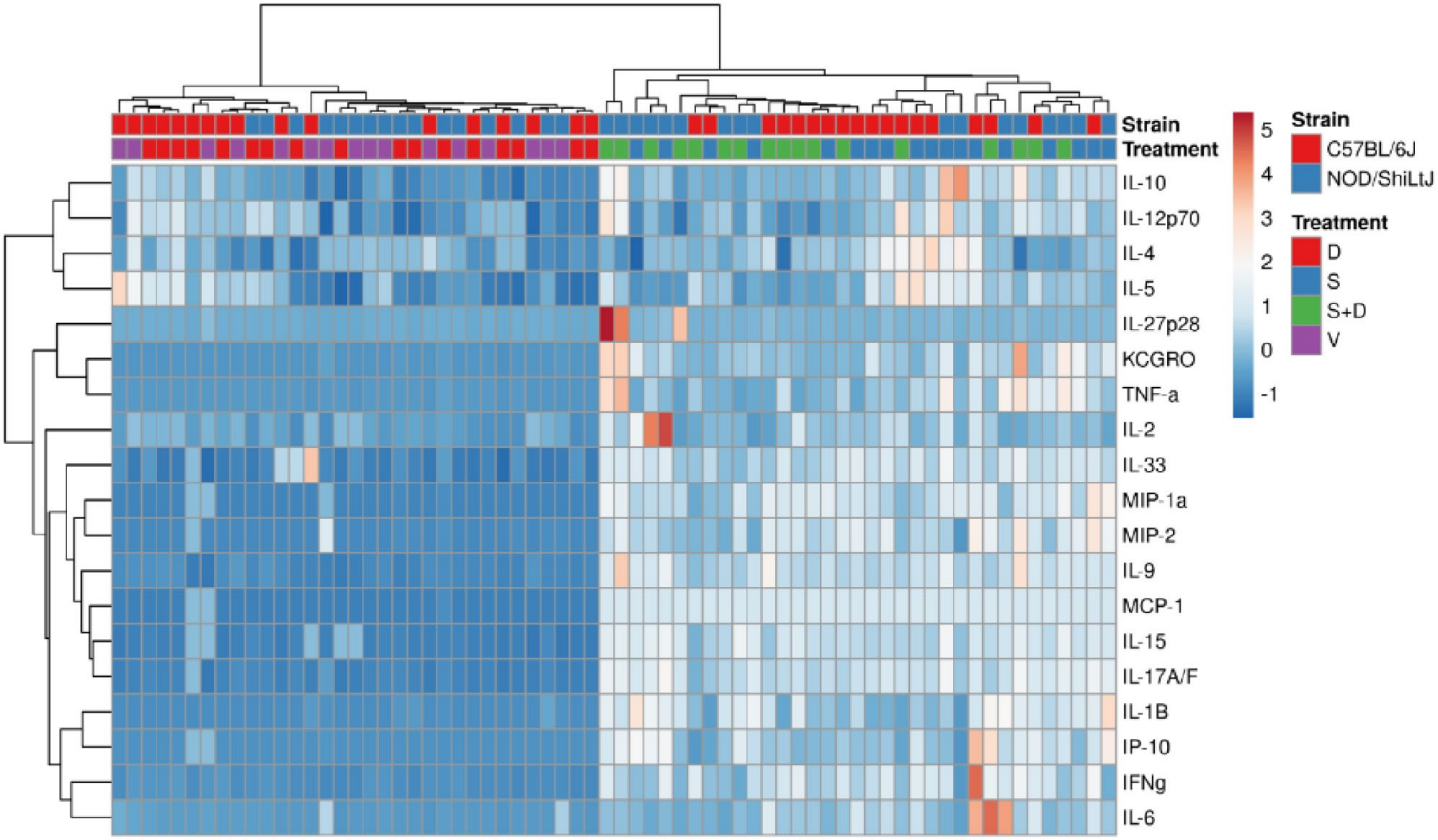
Heatmapping and co-clustering using Euclidean distance and Ward linkage of cytokine and chemokine values (pg/ml) in BAL fluid of vehicle, DEP, silica and silica + DEP exposed C57BL/6J and NOD/ShiLtJ mice. Values were normalized and unit-variance scaled

Hierarchical co-clustering of inflammatory cytokine levels showed how vehicle mice clustered with DEP exposed mice, and how silica mice clustered with silica + DEP exposed mice (Fig. 5). This was further supported by comparing the groups for each cytokine using Two-way ANOVA, showing how almost all the included cytokines and chemokines were upregulated in silica and silica + DEP exposed mice (Additional Fig. 7). The most robust responses in both strains were observed for the macrophage and neutrophil-attracting chemokines MCP-1, MIP-1a, MIP-2, KC/GRO and IP-10 in silica and silica + DEP exposed mice. In addition, pro-inflammatory cytokines related to a Th1 response were upregulated in silica exposed mice in both strains, including IFN-γ and IL-6 (more upregulated in C57BL/6J mice) and IL-15 (more upregulated in NOD/ShiLtJ mice). Additionally, both strains showed an upregulation of IL-1β, which is indicative of the activation of the NLRP3 inflammasome. Moreover, both strains showed an upregulation of IL-33 and IL-9, related to a Th2 response. IL-9 is associated with airway remodeling in the context of asthma, rather than lung inflammation or fibrosis. Finally, both strains showed an upregulation of IL-17 A/F, related to a Th17 response, important in autoimmunity.

Interestingly, within NOD/ShiLtJ mice but not C57BL/6J mice, an additional upregulation of TNF-α, IL-10, IL-12p70 and IL-27p28/IL-30 was detected. Notably, these cytokines all display a regulatory function. IL-10 being an anti-inflammatory cytokine, whereas IL-12p70 forms a link between the innate and adaptive immune system. Nonetheless, it is important to emphasize that the responses were not uniform across all individual mice within a strain.

### BAL fluid antinuclear antibody levels increase upon silica exposure, with stronger responses in NOD/ShiLtJ mice compared to C57BL/6J mice

**Fig. 6:**
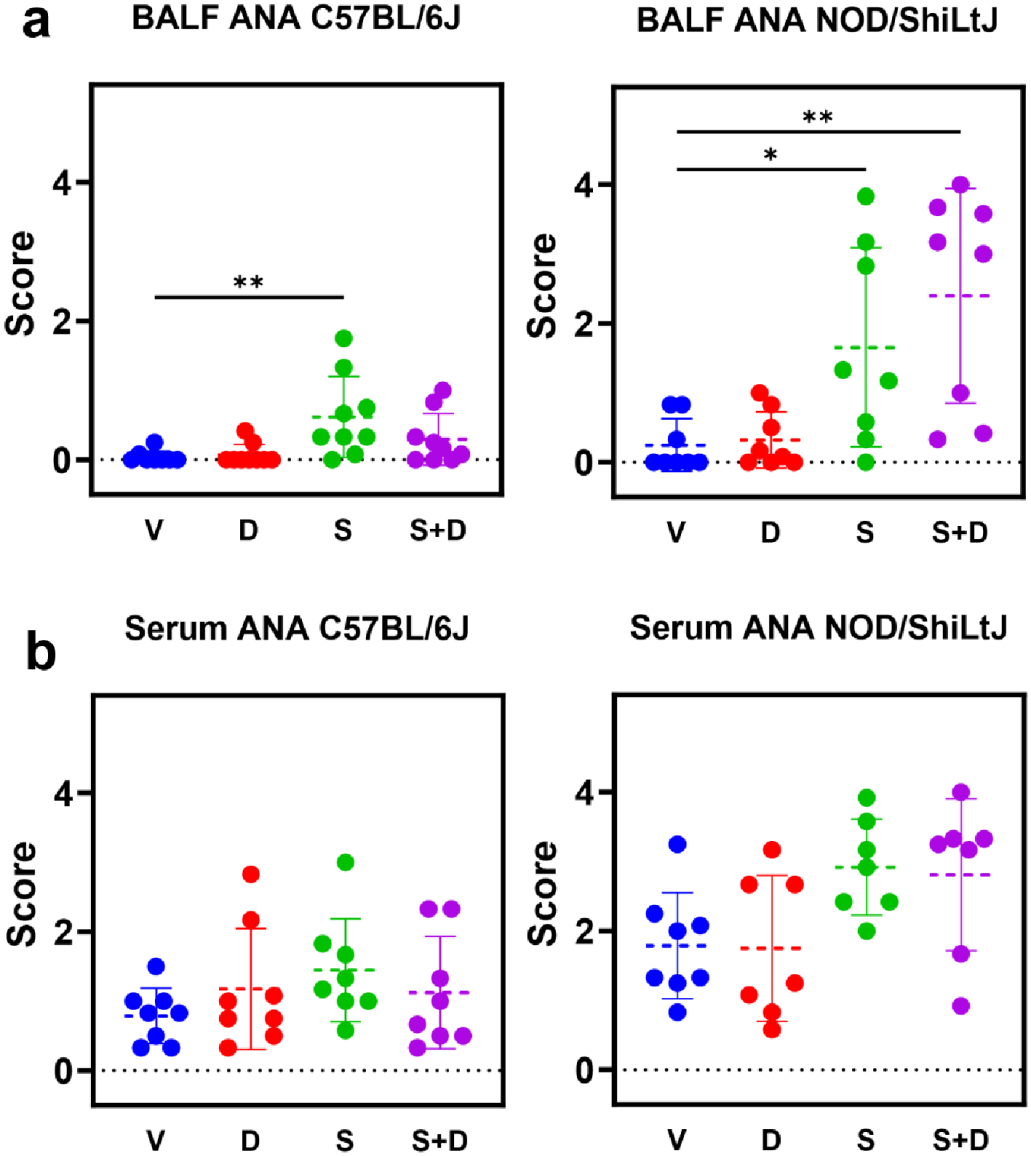
ANA scores based on indirect immunofluorescence assay using HEp2 slides. Scoring was performed by three independent reviewers and averaged for final scores. *N* = 6–9 mice/group. Data are represented as mean ± SD. *p < 0.05, **p < 0.01, ***p < 0.001, ****p < 0.0001 by One-way ANOVA with Tukey correction for multiple testing

To evaluate the development of a local and systemic autoimmune response, antinuclear antibodies (ANA) presence was investigated in BAL fluid and serum. ANA scores in BAL fluid were significantly higher in silica and silica + DEP exposed NOD/ShiLtJ and silica exposed C57BL/6J mice, compared to vehicle and DEP exposed (Fig. 6) mice, with a high variation in responses between individual mice. Anti-nuclear antibody scores in serum were not significantly different between experimental groups. Additionally, vehicle NOD/ShiLtJ mice had higher ANA scores both in serum and BAL fluid compared to vehicle C57BL/6J mice. Again, it is evident that the responses are not uniform across all individual mice within a strain.

### BALF ANA upon silica exposure correlates with extent of lung inflammation in NOD/ShiLTJ mice

To investigate whether we could find correlations between the endpoints within the silica and silica + DEP groups, which were taken together as not significantly different for none of the included endpoints, correlation matrices for C57BL/6J and NOD/ShiLtJ mice were established (Additional Fig. 8). As expected, lung function parameters and micro-CT biomarkers display a strong correlation in both strains. Interestingly, ANA scores in BALF of NOD/ShiLtJ mice correlated negatively with FVC (Pearson *R* = -0.5968, *p* = 0.0188) and IC (Pearson *R* = -0.6662, *p* = 0.0067), indicating that a stronger lung inflammatory response is correlated with a higher extent of ANA in BAL fluid. In C57BL/6J mice, ANA values did not show correlation with lung inflammation biomarkers. In NOD/ShiLtJ, the majority of cytokines and chemokines correlated with each other. However, correlations were less obvious in C57BL/6J mice.

### DEP loaded macrophages and particle co-localization

**Fig. 7:**
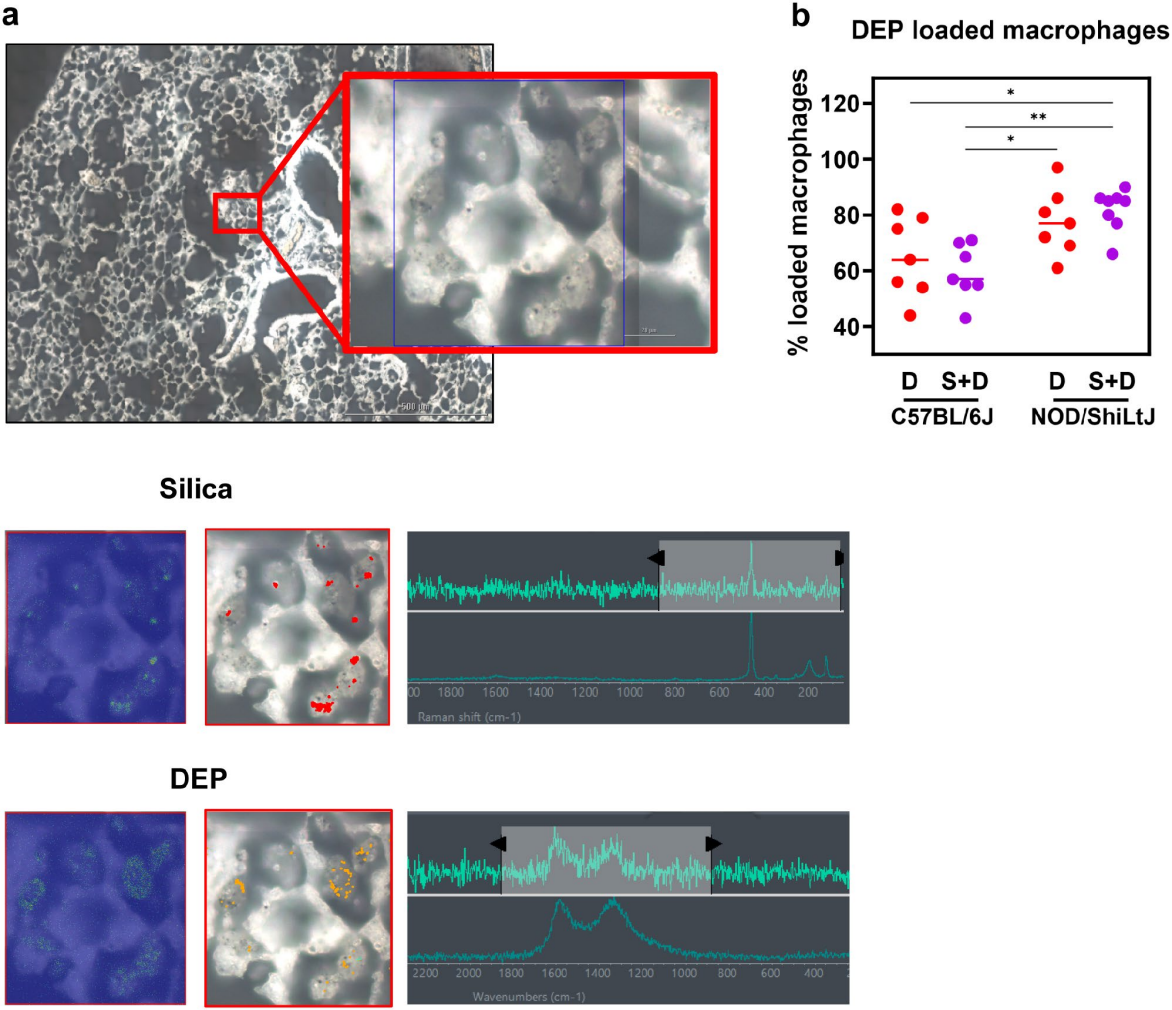
Representative Raman microscopic images of unstained deparaffinized lung section of silica + DEP exposed C57BL/6J mouse; (a) Overview of scanned section showing localization of silica particles, indicated by red dots, and DEP particles indicated by yellow dots, obtained through spectrum identification using OMNIC™xi Software and automatic particle analyzer through library matching (library created using Min-U-Sil 5® and NIST2975 reference materials). (b) % of DEP loaded macrophages in DEP and silica + DEP exposed C57BL/6J and NOD/ShiLtJ mice. Data are represented as mean ± SD. *p < 0.05, **p < 0.01, ***p < 0.001, ****p < 0.0001 by One-way ANOVA with Tukey correction for multiple testing. *N* = 7–8 mice/group

To evaluate particle localization within the lung tissue, lung histological slides were examined using light microscopy for DEP and Raman microscopy for both DEP and silica. The localization of DEP in macrophages was more distinct in Sirius Red-stained lung tissue sections compared to H&E-stained sections. DEP loaded macrophages were observed 10 weeks post last dose in both DEP exposed and DEP + silica exposed C57BL/6J and NOD/ShiLtJ mice. The occurrence of DEP loaded macrophages was notably more pronounced in NOD/ShiLtJ mice exposed to silica + DEP when compared to C57BL/6J mice exposed to DEP and silica + DEP (Fig. 7b). Additionally, in silica + DEP exposed mice, DEP was present in the lung tissue itself compared to almost exclusively in macrophages in DEP-only exposed mice, as observed by qualitative examination. In addition to analyzing the localization of DEP particles through histological examination, we utilized Raman spectroscopy to visualize the co-localization of silica and DEP particles and their uptake by macrophages. In unstained deparaffinized tissue sections, we observed the co-localization of silica and DEP particles within macrophages of silica + DEP exposed C57BL/6J mice (Fig. 7a). These findings highlight the enhanced visualization of DEP localization in macrophages through Sirius Red staining and provide insight into the co-localization of silica and DEP particles within macrophages using Raman spectroscopy on unstained tissue sections.

## Discussion

In this study, we investigated the relationship between lung inflammation, airway hyperreactivity, and antinuclear antibody (ANA) presence in the lungs and systemically upon particulate matter (PM) exposure. We exposed two immunophenotypically distinct mouse strains to silica, DEP or a combination of both. Our aim was to discern variations in pulmonary inflammatory responses at both cellular and cytokine levels, while also assessing clinical implications using lung function measurements and micro-CT. This approach enabled us to establish potential correlations between mouse strain immunophenotypes and their local and systemic autoantibody responses, shedding light on the complex interplay of these factors in pulmonary health. The study design is primarily exploratory, investigating relatively underexplored domains such as combined exposure effects in two immunophenotypically distinct mouse strains. The study’s strength lies in its comprehensive examination of a wide array of outcomes. This extensive analysis offers valuable new insights into these uncharted territories. Given the well-established impact of these particulates on lung function, our study incorporated comprehensive lung function measurements. Furthermore, the diagnostic approach for silicosis in humans often employs micro-CT scans to visualize disease progression. Correspondingly, we adopted micro-CT scans for our murine model, aligning our diagnostic methodology with the clinical standards used in human cases, thereby facilitating a comprehensive evaluation of silicosis development in our experimental context.

A **first objective** was to investigate a possible interaction of silica and DEP exposure on the assessed outcome parameters. Inhalation of PM such as silica and DEP, triggers intricate respiratory responses [30]. Silica particles, deposited in the alveoli and alveolar ducts, activate alveolar macrophages, initiating an inflammatory cascade characterized by pro-inflammatory cytokine release, ultimately leading to chronic inflammation and fibrotic changes, as seen in silicosis [31]. Crystalline silica’s well-documented toxicity results from its crystalline structure and the introduction of surface charge or silanol-containing groups during processing [32, 33]. Reactive oxygen/nitrogen species (ROS/RNS) further sustain lung inflammation. DEP, on the other hand, due to their ultrafine nature, penetrate deep into the lungs, where alveolar macrophages engulf them and release ROS and inflammatory mediators, causing oxidative stress, airway damage, and exacerbating pre-existing respiratory conditions [34]. In addition, they have an exacerbating effect on allergies and asthma by interfering with both exposure and immune response, confirmed both in humans [15, 35] and mice [36]. Unlike silica, DEP are rather linked to chronic obstructive pulmonary disorder (COPD), emphysema, and cancer rather than fibrosis, highlighting the divergent outcomes of DEP exposure compared to silica [37]. The results of all different endpoints consistently show a lack of interaction between the DEP and silica particles in our experimental setup. Moreover, looking at low dose DEP exposure alone, observed adverse effects were limited in the included endpoints. However, low dose DEP exposure independently did elicit subtle yet noteworthy outcomes. Specifically, DEP induced airway hyperreactivity in NOD/ShiLtJ mice, as evidenced by a decline in FEV_0.1_ during methacholine challenge, even at 10 weeks after the last dose. Furthermore, DEP-induced lung inflammation in our study did provoke a discernible inflammatory response in NOD/ShiLtJ mice, detectable by micro-CT. However, with the exception of a slightly higher average macrophage count in DEP exposed mice compared to vehicle exposed mice, most other outcomes, including cytokine levels, did not exhibit differences between the two groups in both strains. This may stem from variations in the sensitivity of the different endpoints employed, with micro-CT proving to be one of the more sensitive and robust measures. Of importance to note is that the used dose of DEP (4 × 10 µg) was chosen to reflect a realistic low exposure dose encountered in daily life, significantly lower than the doses employed in other studies of lung inflammation, with the lowest doses being 25 µg x 3 (total of 75 µg) [38]. Consequently, our lower dose (total of 40 µg) might not have been sufficient to induce additional pronounced effects observed in studies using higher DEP doses. Nevertheless, that makes our findings even more relevant, considering the majority of endpoints were assessed approximately 10 weeks after the last dose. Moreover, DEP particles were still visibly present and detectable using both visual examination of cyto-spins from BAL fluid and Raman spectroscopy on lung tissue slides, also 10 weeks after the last dose. Considering the lack of an interaction between silica and DEP in our study, an aspect to consider is that both exposures involve particulate exposure, which may trigger similar pathways and thereby fail to induce synergistic or significantly exacerbated effects. Moreover, the effects induced by the established dose of silica (4 mg) are large and might dilute out the effects induced by DEP. Further investigations are warranted to elucidate the underlying mechanisms and fully comprehend the observed interactions between DEP and silica in the context of lung effects and fibrosis.

A **second objective** was to investigate the difference between mice species in inflammatory response to silica and/or DEP. Our data showed how NOD/ShiLtJ and C57BL/6J mice respond differently in terms of lung function measurements upon silica and/or DEP exposure, and that these findings were in line with the findings from micro-CT scanning. However, the compensatory mechanism that is more obvious in the C57BL/6J mice, consistent with findings from a study by Dekoster et al. [39] in male C57BL/6J mice, makes it difficult to evaluate which strain develops the worst lung injury and lung function decline in response to silica. The fact that the NOD/ShiLtJ mice did not exhibit an increase in aerated lung volumes following silica exposure, but rather a decrease, is a notable departure from the C57BL/6J model, and more closely resembling what is observed in exposed human subjects. To the best of our knowledge, no studies have been published yet on lung function and lung inflammation after oropharyngeal silica exposure in the NOD/ShiLtJ mouse. Our data suggest that the NOD/ShiLtJ mouse might be a more realistic model of silicosis or silica-induced inflammation compared to the C57BL/6J mouse, especially when aiming to include lung function assessment, as the C57BL/6J mice appear to be resilient for the loss in lung function that comes with the development of silicosis.

NOD/ShiLtJ mice and C57BL/6J mice are known to exhibit significantly distinct baseline immunophenotypic characteristics. C57BL/6J mice were included as a well-studied strain for silicosis and lung inflammation, not spontaneously developing autoimmunity nor developing autoimmune disease upon silica exposure [40]. NOD/ShiLtJ mice, on the other hand, have a chronic inflammatory state, represented by high serum immunoglobulin levels compared to C57BL/6J mice [41]. Reported immune parameters in literature appeared to be worse in female mice, which supports the choice for female mice in the current study [41]. Our study further confirmed this inflammatory state, as mono- and binucleated infiltrates were found in lung tissue sections of vehicle exposed NOD/ShiLtJ mice, which were absent in C57BL/6J mice. NOD/ShiLtJ mice are commonly used as a type 1 diabetes model, as, depending on the conditions, approximately 50–80% of female NOD/ShiLtJ mice spontaneously develop type 1 diabetes. Moreover, these mice also display a propensity for polyautoimmunity, including a low incidence of autoimmune thyroiditis and Sjögren’s syndrome [42]. This was also confirmed in our study, as vehicle exposed NOD/ShiLtJ mice displayed low ANA positivity, while C57BL/6J mice did not exhibit ANA positivity under vehicle exposure. Furthermore, when exposed to heat killed *Mycobacterium bovis*, NOD/ShiLtJ mice exhibit phenotypic features reminiscent of lupus-like autoimmunity [43]. The NOD/ShiLtJ mouse’s autoimmune phenotype lies in the MHC region, specifically in the context of the H-2g7 haplotype. In contrast, C57BL/6J mice exhibit an MHC haplotype, H-2b, which is less or not associated with autoimmunity. The H-2g7 haplotype carried by NOD/ShiLtJ mice is notable for its association with a defect in central tolerance mechanisms, leading to improper negative selection and is essential for the development of type 1 diabetes in these mice [44, 45]. Next to the autoimmune-associated MHC haplotype, the NOD/ShiLtJ mice also bear some other genetic variants impacting immune tolerance, and they exhibit multiple aberrant immunophenotypes including defective antigen presenting cell immunoregulatory functions, defects in the regulation of the T lymphocyte repertoire, defective NK cell function, defective cytokine production from macrophages [46] and impaired wound healing. Therefore, these two strains represent a non-inflammatory, non-autoimmune prone versus an inflammatory phenotype (including polyautoimmunity) concept through which we could take into account the possible influence of genetic background on lung inflammatory and autoimmune features observed.

A **third objective** was to evaluate the response of the two included strains on silica and/or DEP exposure in terms of lung inflammation and function and airway hyperreactivity. Silica inhalation induces local lung damage and the release of damage-associated molecular patterns (DAMPs), which activate the innate immune system through the toll like receptors [47, 48]. This activation leads to the release of several inflammatory mediators, subsequently recruiting macrophages, neutrophils and lymphocytes to the site of injury. Macrophages play a central role in engulfing silica particles, while neutrophils and lymphocytes contribute to the immune response and tissue repair [49]. This response was also observed in our study, in both mouse strains, represented by an increase in mainly neutrophils and macrophages in C57BL/6J mice, and predominantly neutrophils and some lymphocytes in NOD/ShiLtJ mice. It is also remarkable that DEP loaded macrophages (%) were more present in silica + DEP exposed NOD/ShiLtJ mice compared to DEP and silica + DEP exposed C57BL/6J mice, while total cell counts show how C57BL/6J mice had more macrophages in their BAL fluid upon silica exposure compared to NOD/ShiLtJ mice. As there are less macrophages present in the NOD/ShiLtJ mice, the relative DEP load per macrophage will be higher, which could explain the observed results. Furthermore, a wide array of inflammatory cytokines and chemokines was upregulated in BAL fluid of silica exposed mice, with responses being similar between C57BL/6J and NOD/ShiLtJ mice. Although some cytokines were upregulated more in one strain than the other, heatmapping revealed no clear consensus or clusters of cytokines that differed in response between the strains.

As DEP are known to induce airway hyperreactivity (AHR), we included an AHR test using methacholine [50, 51]. The exacerbation of AHR in NOD/ShiLtJ mice by silica exposure suggests the potential of silica exposure on promoting hyperreactivity in the respiratory system, which is confirmed in only few other studies with mice [52]. Silica nanoparticles, however, have been examined more extensively and have been shown to induce AHR [53, 54]. Reports of hyperreactivity in silicosis patients or exposed human subjects, seem to be lacking. One pilot study including 12 silicosis patients demonstrated a normal prevalence of AHR of around 11% [55]. The lack of hyperreactivity reported with silicosis suggests that the AHR upon silica exposure is an effect that only occurs in mouse strains with genetic susceptibility for AHR, such as is observed here to be case with the NOD/ShiLtJ mouse.

While the precise mechanisms linking lung inflammation, silicosis, and autoimmunity remain unclear, recent research has shed light on some key pathways. Chronic exposure to particulates like silica in the lungs can lead to cellular toxicity, tissue damage, inflammation, fibrosis, and the recruitment of autoreactive T and B cells, ultimately culminating in autoimmunity. Notably, silica-induced lung inflammation has been associated with the formation of ectopic lymphoid structures (ELS) within lung tissue, which may contribute to local autoantibody production. However, it’s important to note that this phenomenon appears to be influenced by specific genetic backgrounds. While it is well-documented that silica exposure can induce ANA in lupus-prone strains [40] like NZBWF1/J mice [56], MRL mice [57], BXSB mice [57], and a subset of diversity outbred mice [9], our study represents the first documented case of an exacerbation of the ANA response in NOD/ShiLtJ mice following silica exposure. Furthermore, it is of interest whether worse lung inflammation and lung function are correlated with a higher extent of ANA formation in the lung. Here, it was established that BAL fluid ANA were significantly inversely correlated with FVC and IC in the NOD/ShiLtJ mouse, but not the C57BL/6J mouse, indicating that the processes that determine the intensity of lung function decline are also involved in the processes of local ANA production.

Overall, our study reveals strong discrepancies between C57BL/6J mice and NOD/ShiLtJ mice in terms of lung function and micro-CT biomarkers. The findings suggest that C57BL/6J mice exhibit greater resilience to silica exposure compared to NOD/ShiLtJ mice, likely due to their compensatory increase in aerated lung volume. However, intriguingly, both strains exhibit strikingly similar immune responses at the cellular and cytokine levels when exposed to a high dose of silica. These findings indicate that, despite their contrasting baseline immune profiles, both mouse strains mount a robust and consistent immune reaction to silica exposure. This suggests that additional factors, possibly related to lung morphology or other aspects, may account for the observed differences between NOD/ShiLtJ and C57BL/6J mice [58, 59].

## Conclusion

Our findings strongly support the notion that genetic background, and therefore strain variations, exert a substantial influence on the development of silica-induced lung injury. This underscores the potential value of formal genetic analyses, employing a wider range of strains or recombinant inbred strains derived from these mice. For instance, exploring the Collaborative Cross recombinant inbred strains could prove instrumental in identifying potential loci associated with susceptibility to silica-induced inflammation.

## Methods

### Crystalline silica and Diesel Exhaust Particles (DEP)

Crystalline silica (Min-U-Sil 5®, quartz, CAS: 14808-60-7) was kindly provided by B Fubini (Facoltà di Farmacia, Università di Torino, Italy) and characterized in a previous study [60] using scanning electron microscopy (SEM), which showed fragments, typical of ground silica, ranging from 0.5 to 3 μm. The median size of the particles was about 2 μm as measured by a Coulter LS particle size analyzer at Vlaamse Instelling voor Technologisch Onderzoek (VITO, Belgium) using a Coulter LS particle size analyzer [61]. A more detailed description and results of characterization can be found in Additional File 4.

Diesel particulate matter (Diesel Exhaust Particles, DEPs; Industrial Forklift; NIST2975, CAS: 1333-86-4) was characterized by National Institute of Standards and Technology (NIST, USA) (obtained from Sigma-Aldrich, Bornem, Belgium), for which the particle size ranges from 5.3 to 110 μm. More detailed information on the physico-chemical properties can be found in the safety data sheet provided by Sigma-Aldrich [62].

### Preparation of particle suspensions and doses

Crystalline silica was baked at 200 °C for 1 h to remove endotoxin contamination prior to use. After baking, silica particles were suspended in sterile 0.9% saline + 0.05% Tween at a concentration of 20 mg/ml for silica only exposure (1 mg per dose of 50 µl) and 40 mg/ml for combined (silica + DEPs) exposure (1 mg per dose of 25 µl). DEP were suspended in sterile 0.9% saline + 0.05% Tween at a concentration of 0.2 mg/ml for diesel only exposure (10 µg per dose of 50 µl) and 0.4 mg/ml for combined exposure (10 µg per dose of 25 µl). Fresh suspensions were made for every group and sonicated for 10 min in a bath sonicator to ensure uniform dispersion. The suspension was vortexed immediately before use to obtain a homogeneous suspension.

The DEP dose was selected to reflect the actual human exposure levels to diesel exhaust particles encountered during outdoor physical activities in Flanders, while the crystalline silica dose was selected to reflect occupational exposure over a lifetime. Detailed calculations supporting this concentration are thoroughly explained in Additional File 4.

### Animals

Eight-week-old female NOD/ShiLtJ (*n* = 36) and C57BL/6J (*n* = 36) mice were purchased from Charles River Laboratories (Belgium) and housed 4–5 mice/cage. Mice were housed in a conventional animal facility with 12 h dark/light cycles in individually ventilated cages and were given free access to drinking water and food. Mice were given two weeks of acclimatization before the start of experiments. All experimental procedures were approved by the animal ethics committee of KU Leuven (P111/2021) in compliance with national and European regulations. Background information about the study design and mouse strains can be found in Additional File 3.

### Experimental protocol

For each strain, four experimental groups were included (*n* = 9/group):

- Vehicle (V): control animals were exposed to vehicle only (0.9% saline + 0.05% Tween);
- Silica (S): exposed to 1 mg crystalline silica in 50 µl vehicle;
- Diesel (D): exposed to 10 µg DEPs in 50 µl vehicle; and.
- Silica + DEP (S + D): exposed to both 1 mg crystalline silica and 10 µg DEPs in 50 µl vehicle.

**Fig. 8:**
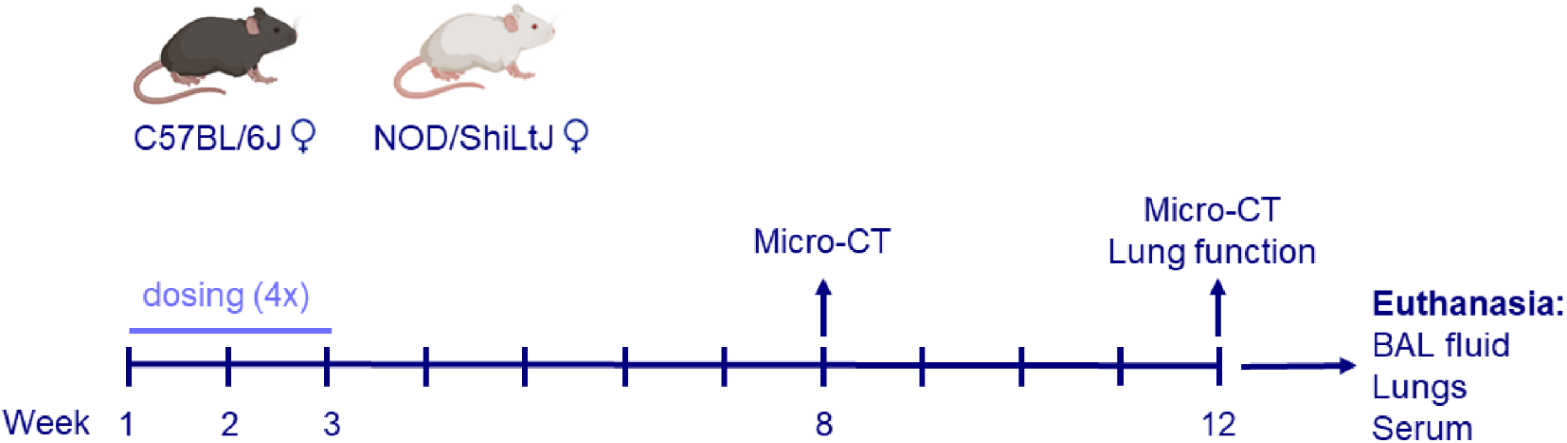
Experimental design. Female C57BL/6J and NOD/ShiLtJ mice were obtained at 8 weeks old. Mice (*n* = 9 per group) were exposed four times over the course of 2 weeks, to either DEP (10 µg in 50 ul per dose), silica (1 mg in 50 µl per dose), both, or vehicle only (50 µl). Micro-CT scans were performed 8 weeks after the start of the experiment and on the day of harvest (12 weeks after start experiment). Lung function measurements using FlexiVent were performed on the day of harvest. Mice were sacrificed and harvested in week 12. BAL fluid was collected for differential cell counts, multiplex cytokine ELISA, and assessment of antinuclear antibodies. Serum was collected for assessment of antinuclear antibodies. Lungs were collected for histopathological assessment, based on formalin fixed paraffin embedded (PFFE) H&E and Sirius Red stained slides

Mice received four doses over the course of two weeks, with two administrations per week, using oropharyngeal aspiration under isoflurane anesthesia, as shown in Fig. 8.

### In vivo lung micro-computed Tomography (µCT)

In brief, mice were anesthetized by inhalation of 1.5-2% isoflurane in oxygen and scanned in supine position using an in vivo µCT scanner (Skyscan 1278, Bruker µCT, Kontich, Belgium) [39]. Scanning parameters and details about the procedure are described in Additional File 4.

### Lung function parameters and airway hyperreactivity

Lung function was assessed using the FlexiVent FX system (SCIREQ, EMKA Technologies, Montreal, Canada), and mice were subsequently euthanized. Measurements were performed as described by Devos et al. [63, 64]. Briefly, the system was designed to measure both forced oscillations (QP3 perturbation) and forced expiration parameters and was operated with FlexiWare™ 7.6 software. The system was equipped with a FX1 module, a negative pressure forced expiration (NPFE) extension for mice, and a small particle size Aeroneb® Lab nebulizer (2.5-4 μm; Aerogen, Galway, Ireland). Mice were anesthetized with pentobarbital (IP, 120 mg/kg body weight, Dolethal®) and once sufficiently anesthetized, a tracheotomy was performed to insert an 18-gauge metal cannula. Mice were quasi-sinusoidally ventilated with a tidal volume of 10 mL/kg and a frequency of 150 breaths/min to mimic spontaneous breathing. At the start of the experiment, two successive deep inflations were applied to maximally inflate the lungs to a pressure of 30 cmH_2_O to open the lungs, and lungs were allowed to equilibrate at that pressure over a period of 3s. The gas compression-corrected volume was read as inspiratory capacity (IC, ml). Airway resistance (Newtonian) (Rn), tissue damping (G) and tissue elastance (H) were assessed using Quick Prime 3. Tissue hysteresivity (G/H) was calculated based on tissue damping and tissue elastance. Forced expiratory volume in 0.1 s (FEV_0.1_), forced vital capacity (FVC) and peak expiratory flow (PEF) were assessed using the NPFE. Tiffeneau-index was calculated based on FEV_0.1_ and FVC (FEV_0.1_/FVC). After performing all perturbations at a baseline level, airway hyperreactivity (AHR) to increasing methacholine concentrations (0, 1.25, 2.5, 5, 10, 20 and 40 mg/ml) [65] was assessed using the forced oscillation technique (QP3 perturbation and NPFE with the same system.

### BAL fluid differential cell counts

Lungs (right and left lobes) were lavaged with 0.7 ml sterile saline (0.9% NaCl) three times in situ. Collected bronchoalveolar lavage fluid (first lavage and the pooled second and third lavage) was centrifuged at 1000 g for 10 min and respective supernatant was stored at − 80 °C. The first lavage supernatant was used for cytokine and anti-nuclear autoantibody (ANA) analyses. Cell pellet was resuspended in 1 ml saline, and 250 µl of the resuspended cells were spun at 300 g for 6 min (Cytospin, 3, Shandon, TechGen, Zellik, Belgium) onto microscope slides, air-dried, and stained (Diff-Quick ® Method, Thermo-Fisher Scientific, Massachusetts, US). A total of 100 cells/animal were manually counted using a light microscope to obtain the ratio of macrophages, eosinophils, neutrophils, and lymphocytes.

To evaluate DEP uptake by alveolar macrophages, DEP loaded macrophages were counted using BAL fluid cytospin slides. The percentage of loaded macrophages was determined by manually counting a total of 100 macrophages using a light microscope (Additional Fig. 9).

### Cytokine and chemokine levels in BAL fluid

Cytokine and chemokine levels were determined in undiluted BAL fluid supernatant using the V-PLEX® Proinflammatory and Cytokine Panel 1 (mouse) Kit MSD® Multi-Spot Assay System (Meso Scale Diagnostics, LLC), according to protocol. Absorbance was measured on the Meso Scale Discovery (MSD) plate reader (Meso Scale Diagnostics, Maryland, USA). The following cytokines and chemokines were included in the panel: IL-4, IL-10, IFN-γ, IL-2, IL-5, KC/GRO, IL-1β, IL-12p70, TNF-α, IL-6, IL-15, IP-10, MCP-1, MIP-1α, IL-9, IL-17 A/F, IL-33, IL-27p28/IL-30 and MIP-2. Detection limits can be found in Additional File 4.

### BAL fluid and serum anti-nuclear antibodies using Indirect immunofluorescence (IIF)

Antinuclear antibody (ANA) presence was evaluated in the supernatant of 1:10 diluted BAL fluid and 1:100 diluted serum samples using NOVA Lite® HEp-2 ANA slides (Inova Diagnostics). The experimental procedure involved applying the diluted samples (1:10) onto HEp-2 cell-containing slides and incubating them for 1 h at 21 °C. Subsequently, slides were washed to eliminate unbound antibodies and immersed in phosphate-buffered saline (PBS) for 5 min. Detection of bound antibodies was accomplished by incubating slides with goat anti-mouse IgG Alexa Fluor 488 (Southern Biotech, 1030-30) diluted in 0.05% PBS-Tween (1:400) for 1 h at 21 °C. Following another wash in 0.05% PBS-Tween for 5 min, slides were covered with a coverslip. For the semi-quantitative assessment of fluorescence intensity, two representative images per mouse were acquired. The evaluation was conducted by three independent scorers (LJ, FL, NH) in a blinded manner. A scoring system, as described by the manufacturer, was employed to evaluate the intensity of the fluorescence, as follows:

(0) Intensity comparable to the negative control, indicating no discernible fluorescence.

(1+) Lowest fluorescence intensity, with a distinct demarcation between background fluorescence and nuclear and/or cytoplasmic fluorescence.

(2+) Clearly distinguishable positive fluorescence.

(3+) Similar intensity to the positive control.

(4+) Brilliant apple green fluorescence, exhibiting a brighter intensity compared to the positive control.

Each scorer independently assigned a score to the observed fluorescence intensity, ensuring consistency and minimizing bias. The final intensity score for each sample was determined by averaging the scores assigned by the three scorers.

### Lung histopathology and particle colocalization

After lavage, the left lung lobe was filled with 4% formaldehyde and tied off, removed from the body and immersed in 4% formaldehyde for fixing (at least 48 h), whereafter formaldehyde was replaced with 70% ethanol. Paraffin embedded tissue Sect. (5 μm) were stained with Hematoxylin and Eosin (H&E) for general cellular and tissue morphology and Sirius Red for the presence of collagen fibers. Adjacent sections were used for the two different staining. Sections were blindly examined by a professional pathologist (AV) using light microscopy. In addition, a standardized grading scale, the modified Ashcroft scale, was used to grade pulmonary fibrosis in H&E-stained sections by two separate scorers (FL, MG). Briefly, five fields of H&E-stained lung tissue were inspected using a 20-fold objective. Each field received a grade from 0 to 8, based on short descriptions of alveolar septa and lung structure and mostly based on reference images [29]. Grades were added up and divided by the number of fields to obtain a fibrotic index (FI) for each mouse per group (*n* = 4 mice/group). Grades from scorers were averaged to obtain a final grade ± SD for each experimental group.

To detect and qualitatively examine colocalization of silica and DEP particle deposition in alveolar macrophages, particles inside the tissue were visualized in an unstained deparaffinized section using Dxr3xi Raman imaging microscope (Thermo Fisher Scientific; Scan setting- Laser Power 2 mW, exposure time 0.01 Sect. (100 Hz), number of scans = 8, image pixel size 0.2 μm). One section for each experimental group was scanned of the C57BL/6J mice.

### Statistical analyses and data visualization

Scatter plots and stacked columns were created in GraphPad/Prism (Graphpad Software version 9.3.1, La Jolla, CA, https://www.graphpad.com/) representing mean ± SD, unless mentioned otherwise. Experimental groups were compared using One-way ANOVA within strains, or Two-way ANOVA with repeated measures for outcomes including two timepoints. Cytokine values were compared using Two-way ANOVA. Tukey’s multiple comparisons test was used for further comparisons between groups. To compare responses to DEP and/or silica between strains, fold changes over vehicle were calculated and values were compared using multiple t-tests with Holm-Šídák method for multiple testing. *p* < 0.05 was considered statistically significant, and levels of significance were indicated as follows; *p < 0.05, **p < 0.01, ***p < 0.001 and ****p < 0.0001. Correlation matrices were created using the Corrplot package in R (R Core Team, 2023).Heat mapping and hierarchical co-clustering (HCC) were performed using ClustVis online software [66]. Normalized and unit variance-scaled raw values were represented in heat maps, with data organized by unsupervised HCC. Values were centered by rows; imputation was used for missing value estimation. Rows and columns were clustered using Euclidean distance and Ward linkage.

### Electronic supplementary material

Below is the link to the electronic supplementary material.

Vehicle micro-CT comparisons

Lung densities based on micro-CT

Transverse micro-CT sections

Supplemental FlexiVent data

DEP uptake by macrophages

SR staining of lung histology

Correlation Matrices

Absolute BAL fluid cell counts

DEP uptake by macrophages

Strain-dependent differences in response

Qualitative observations lung histology

Background information study design and mouse strains

Additional information methods

## Data Availability

The datasets analyzed during the current study are available from the corresponding author upon reasonable request.
