# Supplementary figures and images for: Differential pulmonary toxicity and autoantibody formation in genetically distinct mouse strains following combined exposure to silica and diesel exhaust particles
Source: Part Fibre Toxicol. 2024 Feb 27;21:8. doi: 10.1186/s12989-024-00569-7 (PMC10898103; doi:10.1186/s12989-024-00569-7)

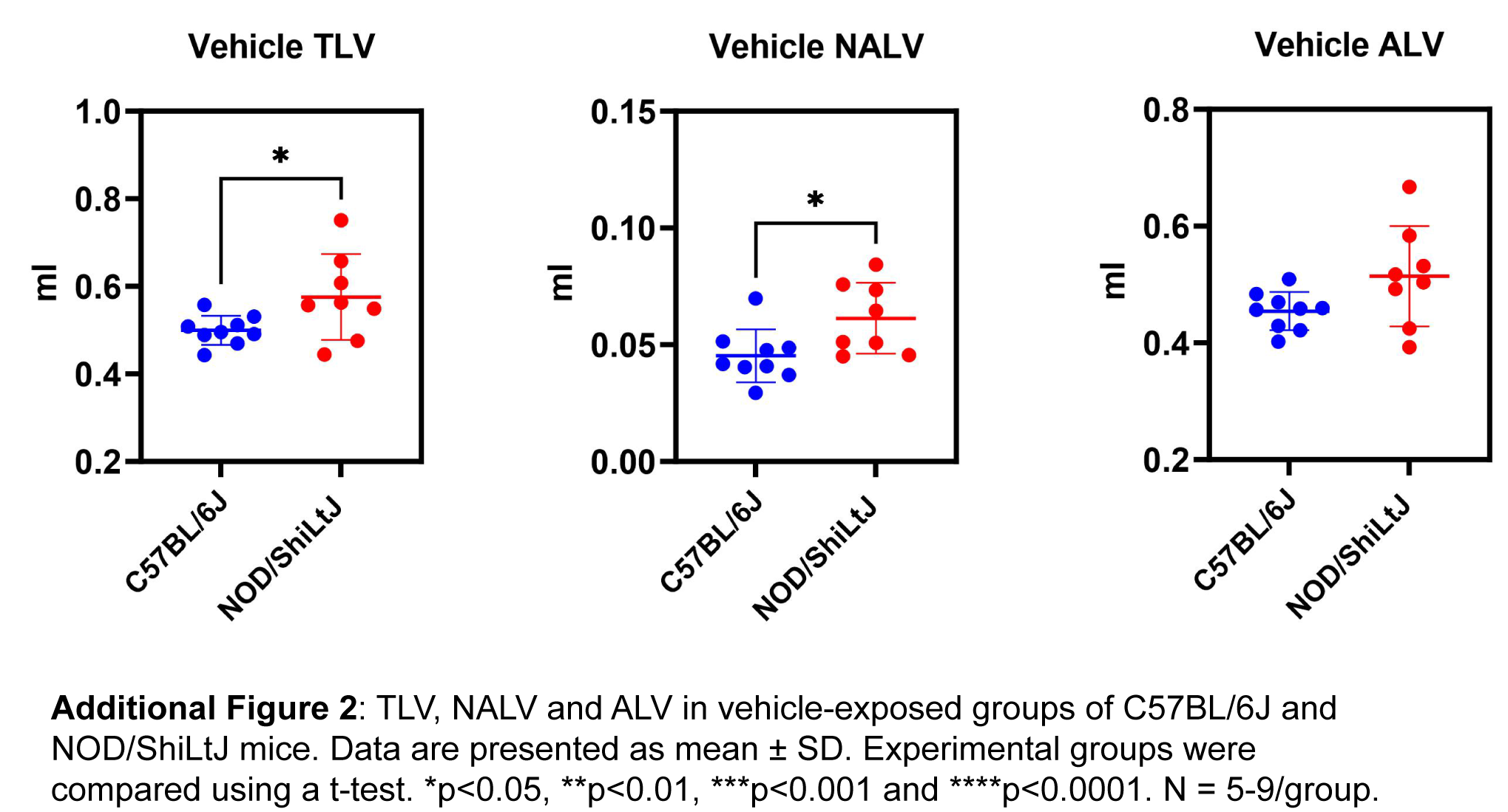

Supplement: Supplementary file 1 — Vehicle micro-CT comparisons [file 12989_2024_569_MOESM1_ESM.tif]

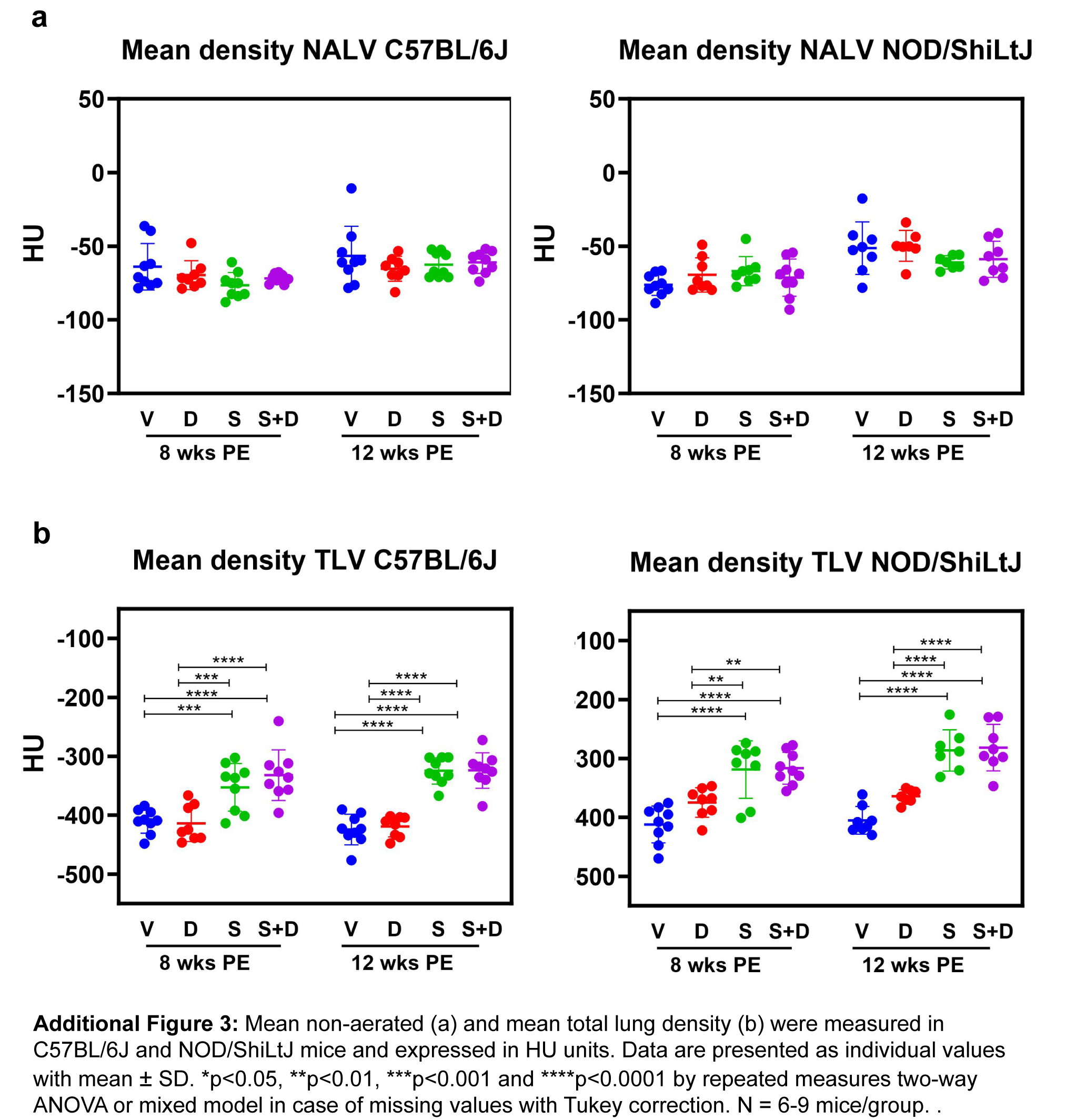

Supplement: Supplementary file 2 — Lung densities based on micro-CT [file 12989_2024_569_MOESM2_ESM.tif]

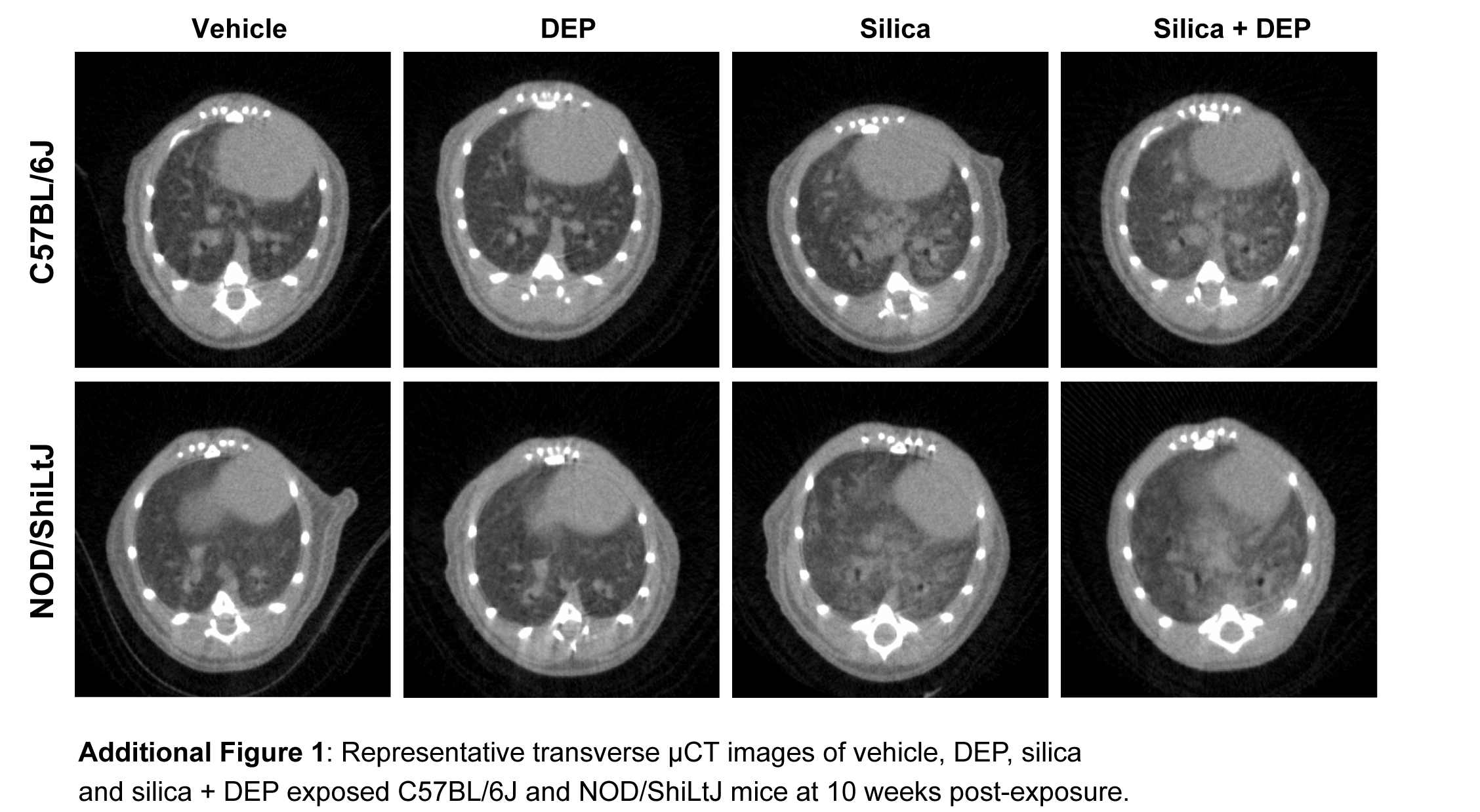

Supplement: Supplementary file 3 — Transverse micro-CT sections [file 12989_2024_569_MOESM3_ESM.tif]

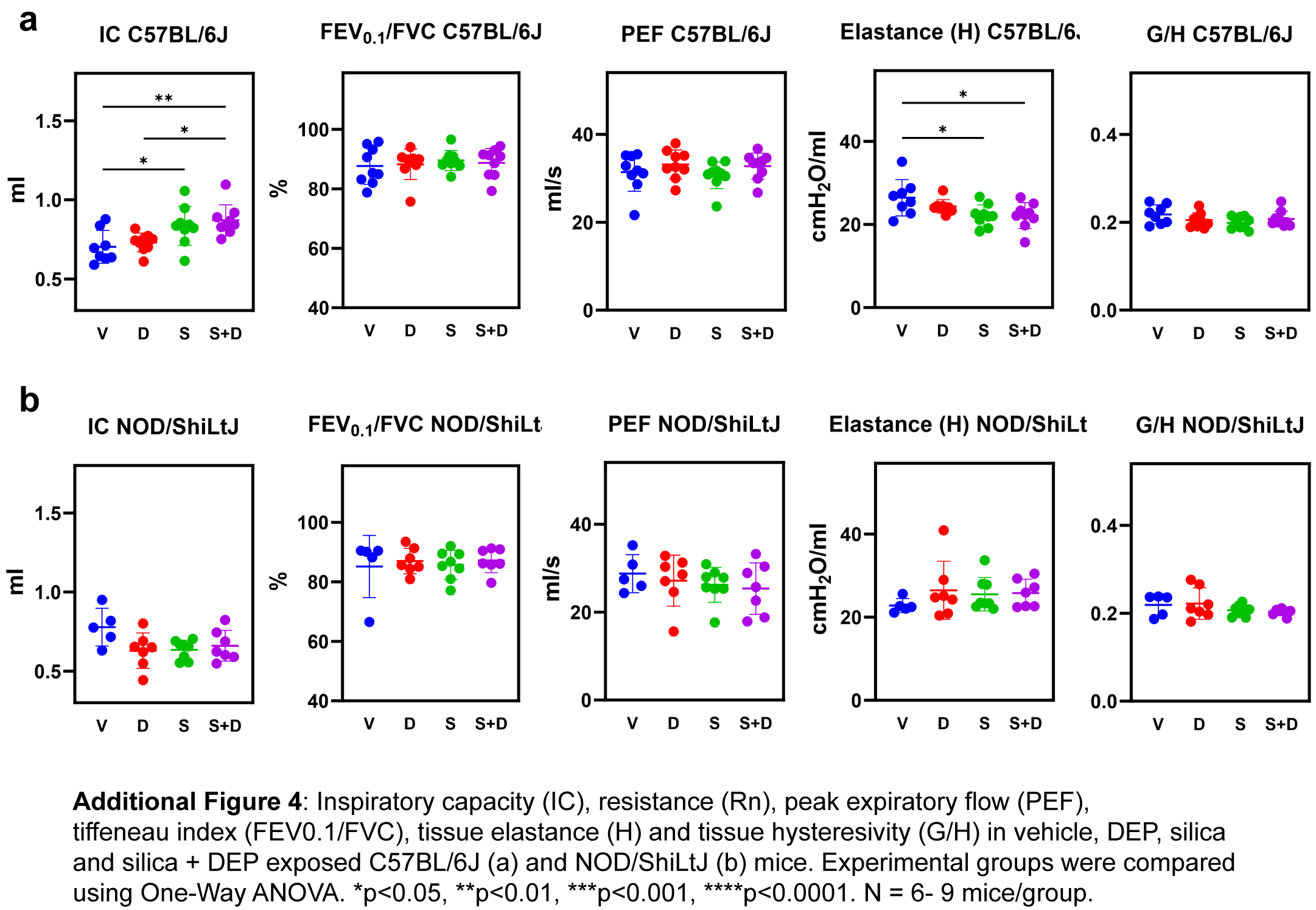

Supplement: Supplementary file 4 — Supplemental FlexiVent data [file 12989_2024_569_MOESM4_ESM.tif]

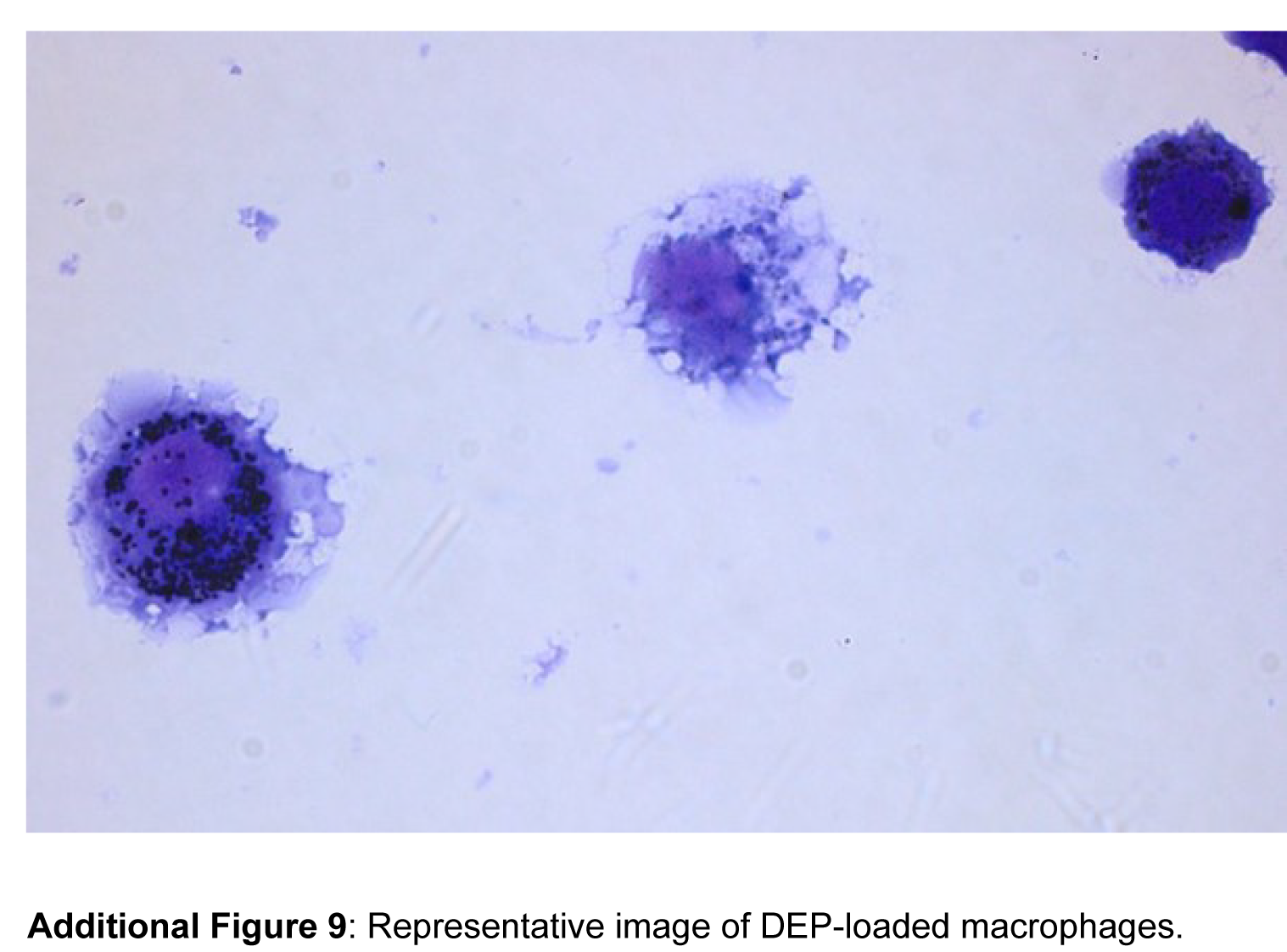

Supplement: Supplementary file 5 — DEP uptake by macrophages [file 12989_2024_569_MOESM5_ESM.tif]

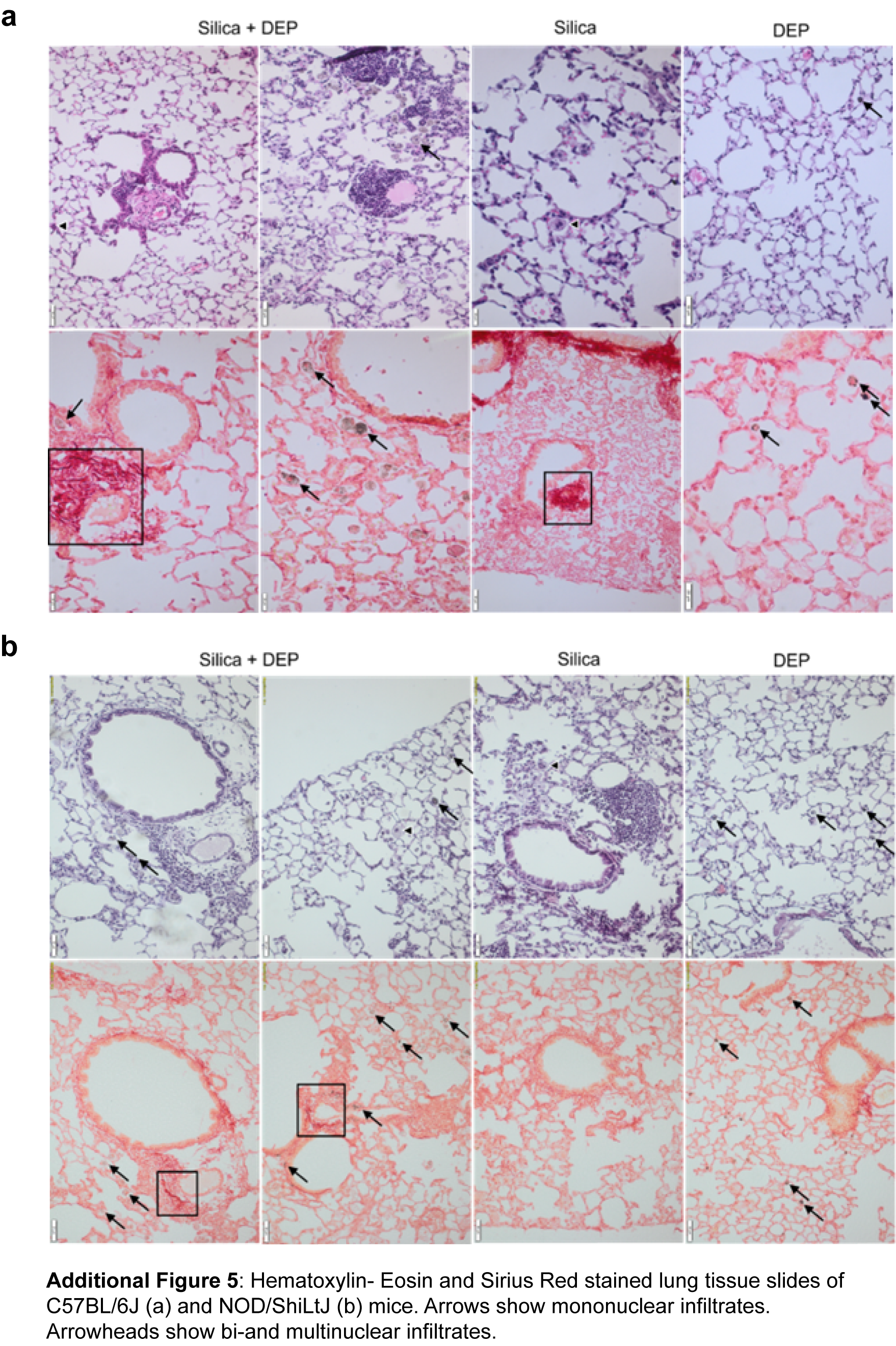

Supplement: Supplementary file 6 — SR staining of lung histology [file 12989_2024_569_MOESM6_ESM.tif]

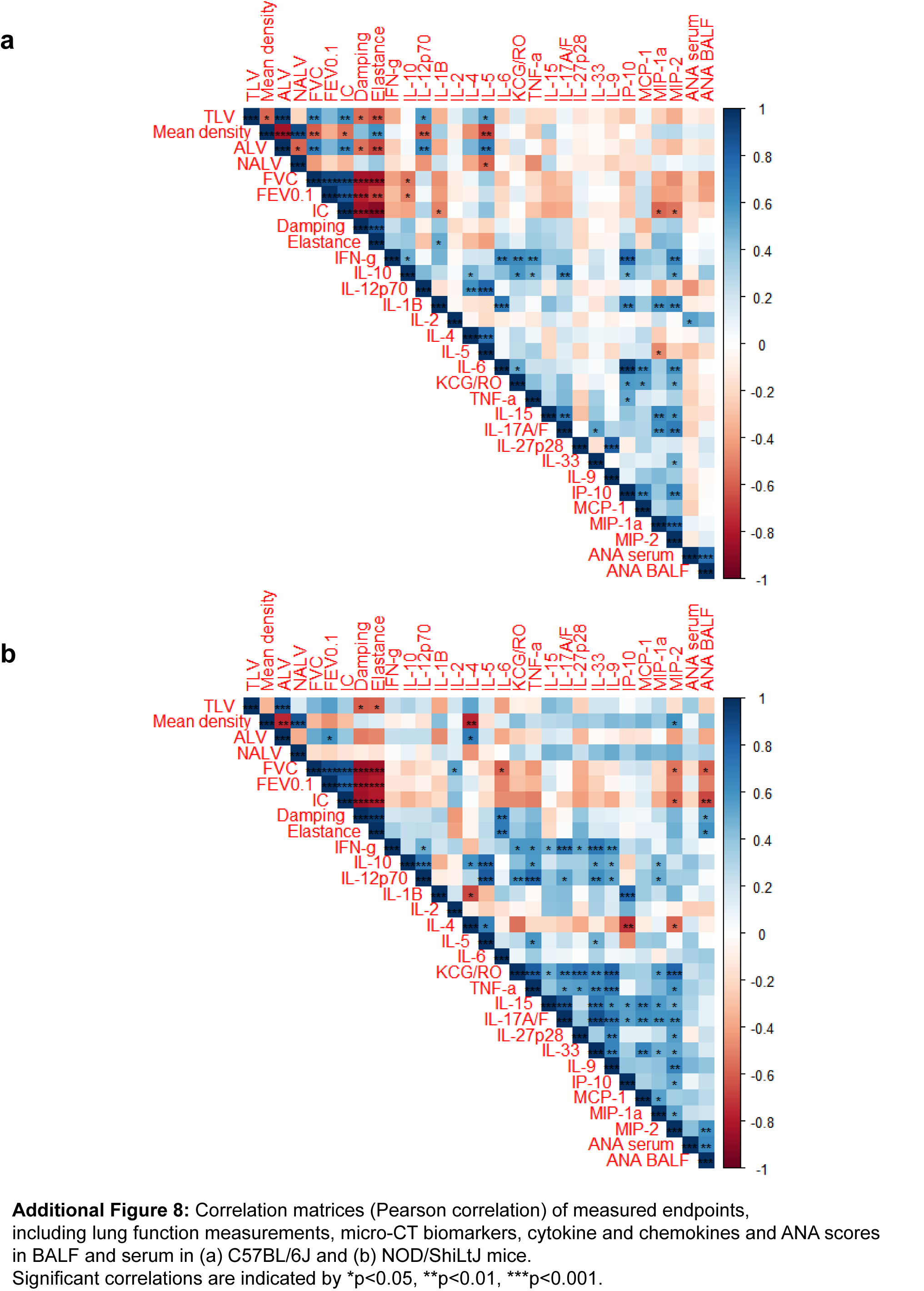

Supplement: Supplementary file 7 — Correlation Matrices [file 12989_2024_569_MOESM7_ESM.tif]

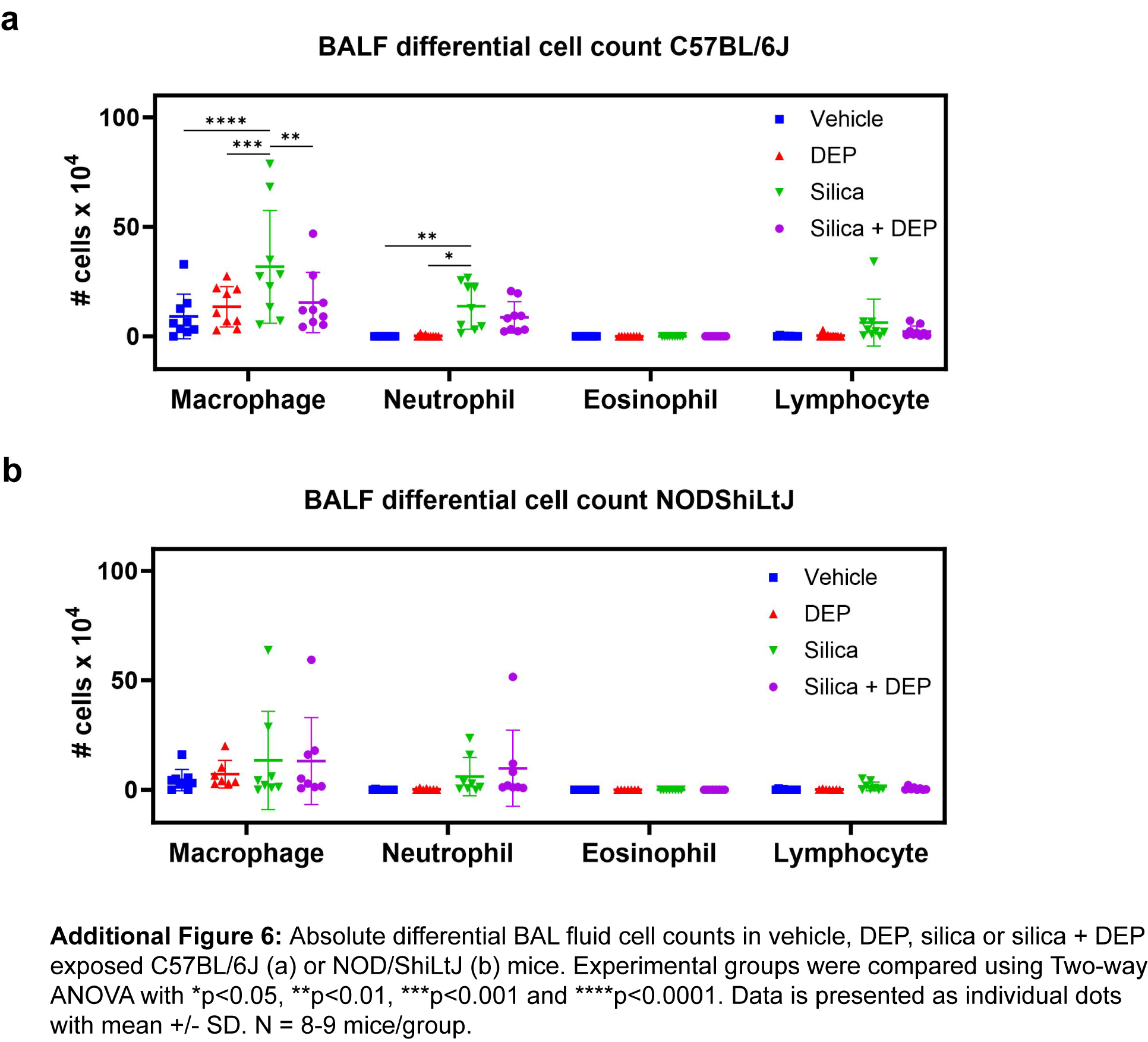

Supplement: Supplementary file 8 — Absolute BAL fluid cell counts [file 12989_2024_569_MOESM8_ESM.tif]

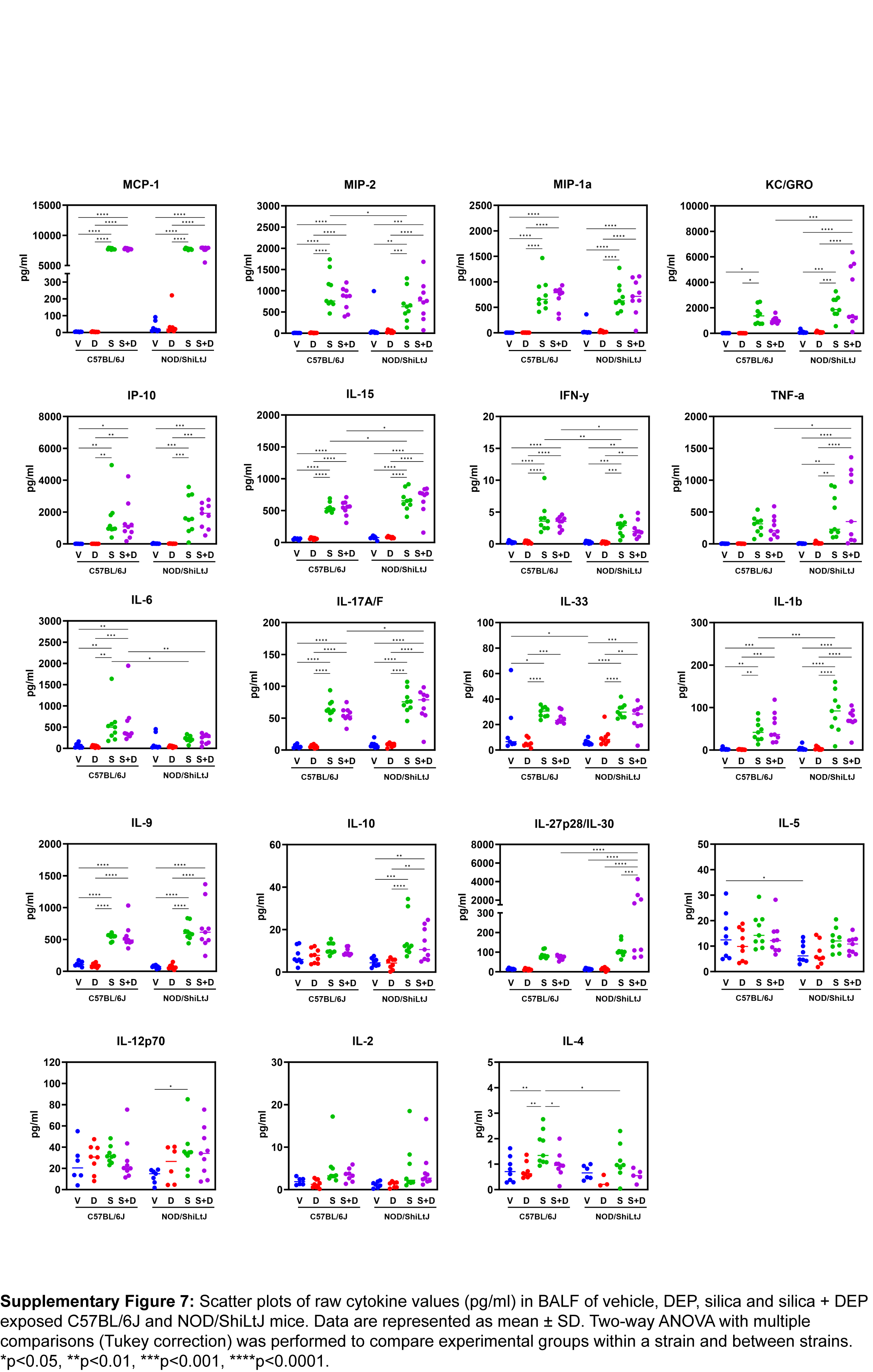

Supplement: Supplementary file 9 — DEP uptake by macrophages [file 12989_2024_569_MOESM9_ESM.tif]
