# Supplementary material for: Differential pulmonary toxicity and autoantibody formation in genetically distinct mouse strains following combined exposure to silica and diesel exhaust particles
Source: Part Fibre Toxicol. 2024 Feb 27;21:8. doi: 10.1186/s12989-024-00569-7 (PMC10898103; doi:10.1186/s12989-024-00569-7)
Supplement: Supplementary file 10 — Strain-dependent differences in response [file 12989_2024_569_MOESM10_ESM.docx]

|  | D | | | S | | | S+D | | |
| --- | --- | --- | --- | --- | --- | --- | --- | --- | --- |
|  | C57BL/6J | NOD/ShiLtJ |  | C57BL/6J | NOD/ShiLtJ |  | C57BL/6J | NOD/ShiLtJ |  |
| NALV | 1.019 | 1.156 | ns | 3.176 | 3.085 | ns | 3.127 | 3.034 | ns |
| ALV | 1.051 | 0.9369 | ns | 1.262 | 1.024 | * | 1.227 | 0.9743 | ** |
| ALV density | 0.9886 | 0.9208 | * | 0.8228 | 0.8225 | ns | 0.8151 | 0.8121 | ns |
| FVC | 1.062 | 0.9085 | ** | 1.122 | 0.8678 | ** | 1.144 | 0.8748 | *** |
| FEV_0.1_ | 1.067 | 0.9143 | ** | 1.145 | 0.8582 | *** | 1.156 | 0.8834 | **** |
| IC | 1.039 | 0.8094 | ** | 1.189 | 0.8161 | *** | 1.239 | 0.8481 | *** |
| PEF | 1.056 | 0.9451 | ns | 0.9777 | 0.9113 | ns | 1.044 | 0.882 | ns |
| FVC/FEV_0.1_ | 1.006 | 1.021 | ns | 1.021 | 1.006 | ns | 1.012 | 1.024 | ns |
| Rn | 0.9915 | 1.093 | ns | 0.918 | 1.328 | * | 0.8787 | 1.196 | ** |
| G | 0.8665 | 1.154 | ** | 0.7641 | 1.063 | ** | 0.7891 | 1.055 | *** |
| H | 0.9219 | 1.161 | * | 0.8375 | 1.12 | ** | 0.8368 | 1.132 | ** |
| G/H | 0.9401 | 0.7881 | ns | 0.9118 | 0.8385 | ns | 0.9519 | 0.7212 | ns |
| Lung fibrosis score | 0.8958 | 0.8033 | ns | 1.995 | 2.107 | ns | 1.833 | 1.393 | ns |
| Macrophages | 0.9556 | 1 | ns | 0.6589 | 0.9739 | *** | 0.6106 | 0.5914 | ns |
| Neutrophils | 2.222 | 2.857 | ns | 56.44 | 18.94 | * | 64.67 | 20.76 | **** |
| Lymphocytes | 0.8081 | 0.8 | ns | 3.192 | 5.796 | ns | 3.071 | 3.492 | ns |
| ANA serum | 1.489 | 0.9777 | ns | 1.832 | 1.63 | ns | 1.422 | 1.57 | ns |
| ANA BALF | 2.03 | 1.296 | ns | 15.88 | 6.653 | ns | 8.061 | 9.633 | ns |

**Additional File 1**

Comparison in responses between C57BL/6J and NOD/ShiLtJ mice to DEP, silica and silica + DEP. Expressed as fold changes over vehicle-group. Multiple t-tests with Holm-Šídák method for multiple testing was applied to assess significant differences between responses. Table consists of mean values with indication of significance levels (*p<0.05, **p<0.01, ***p<0.001, ****p<0.0001).
