# Supplementary material for: Differential pulmonary toxicity and autoantibody formation in genetically distinct mouse strains following combined exposure to silica and diesel exhaust particles
Source: Part Fibre Toxicol. 2024 Feb 27;21:8. doi: 10.1186/s12989-024-00569-7 (PMC10898103; doi:10.1186/s12989-024-00569-7)
Supplement: Supplementary file 11 — Qualitative observations lung histology [file 12989_2024_569_MOESM11_ESM.docx]

**Additional File 3**

Background information on study design and course

To define the number of animals needed in each group, a power analysis using the BioMath online tool (http://biomath.info/power/) was performed based on a t-test on group means. The measurement of autoantibodies was used as endpoint to estimate the number of animals needed for the experiments, since the immune phenotyping will mainly be based on this measurement. Based on papers included in our published systematic review on exposure to silicates and autoimmunity outcomes in rodents (Exposure to silicates and systemic autoimmune-related outcomes in rodents: a systematic review, 10.1186/s12989-021-00439-6), the average number of mice per group is n = 7. In a paper published by our group (Choice of mouse strain influences the outcome in a mouse model of chemical-induced asthma, 10.1371/journal.pone.0012581.), comparing 7 mouse strains reporting several immunological endpoints, the number of animals per group ranged from 4 – 9. Finally, based on these papers and the power analysis, we concluded that n = 9 mice per group should be sufficient. As exclusion criterium, during the course of the experiment, a body weight reduction of at least 20% was used as a basis to prematurely exclude/sacrifice a mouse. On the basis of this, only one mouse (diesel-exposed NOD/ShiLtJ mouse) was prematurely excluded during the experiment. This mouse was sacrificed 2 weeks before the end of the experiment. At several points during the experiment, we randomized our choice of mice. First of all, mice were randomly divided over cages at the start of the experiment in order to make groups of 9. Furthermore, at the day of dosing they were then picked out in a random order per group in order to receive their exposure.

Background information of mouse strains

Source : <https://www.jax.org/strain/000664>

C57BL/6J mice are an inbred strain with a homozygous a/a genotype. They are widely employed as a general-purpose strain in scientific research. They possess several desirable characteristics, including good breeding performance, longevity, and a low susceptibility to tumors. Furthermore, they are resistant to audiogenic seizures, have a relatively low bone density and are prone to age-related hearing loss. They are also susceptible to diet-induced obesity, type 2 diabetes, and atherosclerosis. Notably, macrophages derived from C57BL/6J mice demonstrate resistance to the effects of anthrax lethal toxin.

Source : <https://www.jax.org/strain/001976>

NOD/ShiLtJ mice are an inbred strain with related genotype (A/A Tyrc/Tyrc). They represent a polygenic model for autoimmune type 1 diabetes. The presence of diabetes in NOD mice is characterized by hyperglycemia and insulitis. Marked decreases in pancreatic insulin content occur in females at about 12 weeks of age and in males this is typically several weeks later. A 2022 phenotyping study found that 86% of females and 48% of males became diabetic by 30 weeks of age; median female incidence was 18 weeks. Immune phenotypes in the NOD background consist of defects in antigen presentation, T lymphocyte repertoire, NK cell function, macrophage cytokine production, wound healing, and C5 complement.
