# Supplementary material for: Differential pulmonary toxicity and autoantibody formation in genetically distinct mouse strains following combined exposure to silica and diesel exhaust particles
Source: Part Fibre Toxicol. 2024 Feb 27;21:8. doi: 10.1186/s12989-024-00569-7 (PMC10898103; doi:10.1186/s12989-024-00569-7)
Supplement: Supplementary file 12 — Background information study design and mouse strains [file 12989_2024_569_MOESM12_ESM.docx]

**Additional File 2**

Lung histology detailed observations

**Table 1:** Main pathological findings based on H&E-stained lung tissue slices.

|  | **C57BL/6J** | **NOD/ShiLtJ** |
| --- | --- | --- |
| **Vehicle** | Normal histology | More diffuse inflammatory infiltrates (compared to C57BL/6J)  Presence of macrophages |
| **DEP** | Minimal diffuse inflammatory infiltrates | More diffuse inflammatory infiltrates (compared to C57BL/6J) |
| **Silica** | More and bigger mononuclear inflammatory infiltrates (compared to DEP and Vehicle) | More diffuse inflammatory infiltrates (compared to C57BL/6J) |
| **Silica + DEP** | More and bigger mononuclear inflammatory infiltrates (compared to DEP and Vehicle) | More diffuse inflammatory infiltrates (compared to C57BL/6J) |

**Table 2:** Main pathological findings based on SR-stained lung tissues slices.

|  | **C57BL/6J** | **NOD/ShiLtJ** |
| --- | --- | --- |
| **Vehicle** |  |  |
| **DEP** | DEP-loaded macrophage and DEP in tissue | DEP-loaded macrophage and DEP in tissue |
| **Silica** | Bi- or mononucleated cells  More collagen deposition (compared to vehicle and DEP) | Bi- or mononucleated cells  More collagen deposition (compared to vehicle and DEP) |
| **Silica + DEP** | Bi- or mononucleated cells  More collagen deposition (compared to vehicle and DEP)  DEP-loaded macrophage and DEP in tissue | Bi- or mononucleated cells  More collagen deposition (compared to vehicle and DEP)  DEP-loaded macrophage and DEP in tissue |
