# Supplementary material for: Differential pulmonary toxicity and autoantibody formation in genetically distinct mouse strains following combined exposure to silica and diesel exhaust particles
Source: Part Fibre Toxicol. 2024 Feb 27;21:8. doi: 10.1186/s12989-024-00569-7 (PMC10898103; doi:10.1186/s12989-024-00569-7)
Supplement: Supplementary file 13 — Additional information methods [file 12989_2024_569_MOESM13_ESM.docx]

**Additional File 4**

Particle information

*Crystalline silica particles:*

Samples for electron microscopy were dispersed in ultrafiltered ethanol, using a vortex mixer (1). Droplets of the resulting suspension were applied to aluminum specimen holders, dried-down, sputter-coated with gold, and examined in an ISI DS130 scanning electron microscope. The remaining suspension was mixed (1 + 1) with ultrafiltered, ultrapure water and mixed again. Droplets of this suspension were applied to nickel grids, precoated with a film of formvar/carbon, and examined in a Zeiss 902A transmission electron microscope. The samples of Min-U-Sil contained fragments typical of ground silica, mostly ranging from 0.5–3.0 µm in the largest dimension. See below for scanning electron micrograph showing the surface morphology:


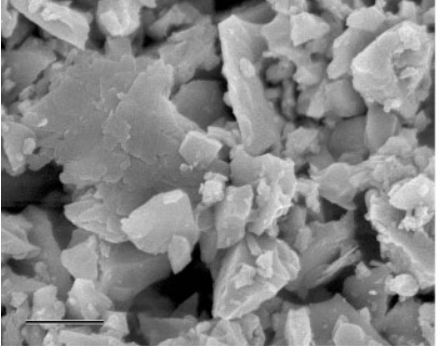


Figure is copied from (1).

Information and calculations supporting used particulate matter doses

*Diesel exhaust particles:*

To demonstrate the applicability of our chosen DEP exposure concentration in the model, we estimated the amount of DEP inhaled by humans during a typical 30-minute session of light exercise (with a minute volume of 18 L/min) in an environment with a PM2.5 concentration of 14.5 μg/m3. This estimation is based on the 14-day average PM2.5 level recorded in Leuven, Belgium, as reported by the Intergewestelijke Cel voor het Leefmilieu (IRCEL) (2). The calculated human exposure equates to approximately 7.5 μg PM2.5, aligning with the concentration range used in our mouse model. Additionally, this concentration is consistent with the World Health Organization's (WHO) limit values of 25 μg/m^3^ (3). Our analysis underscores the relevance of our study's findings to everyday air pollution exposure scenarios in Flanders, representing a significant advantage of our research approach.

*Crystalline silica:*

The established dosage of crystalline silica (Min-U-Sil5) for inducing silicosis and autoimmunity in animal research is well-documented in studies to be 4-5 mg (4, 5). Additionally, the relevance of this silica dosage to real-world human exposure merits consideration. Our study utilizes calculations from Rajasinghe et al. (5) as a basis. The U.S. Occupational Safety and Health Administration (OSHA) sets the permissible exposure limit (PEL) for respirable crystalline silica at 50 mg/m3/day, as detailed on their website (<https://www.osha.gov/Publications/OSHA3682.pdf>). Many industries, including demolition, crushing, chipping, and fracking, frequently exceed this limit (6, 7). With an estimated human ventilation rate of 6.0 L/min, exposure to the OSHA PEL could lead to the inhalation of 1433 mg of silica over a 40-year career (8 hours a day, 5 days a week). In contrast, mice with an estimated ventilation rate of 0.03 L/min would be predicted to have an equivalent lifetime exposure of 8.28 mg of silica. Therefore, the total silica doses used in our study, 4 mg, represent approximately half the human lifetime exposure to silica at the OSHA's recommended limit.

Transoral instillation

In brief, anaesthetized mice were suspended by the cranial incisors on a thin thread from a plastic slanted intubation stand and fixed in position with a piece of tape. The tongue was gently extracted from the mouth using blunt forceps and held between two fingers to visualize the base of the tongue and the pharynx. Particle suspension or vehicle was placed in the posterior pharynx with a micropipette, and nares were gently pinched with blunt forceps. Respiration was monitored to ensure that the suspension was fully aspirated before the tongue and nares were released. Mice were allowed to recover until fully awake in a separate cage. General health of the mice was assessed by monitoring weight weekly and visual check-ups based on fur condition, posture, and behavior.

In vivo micro-CT scanning

The following parameters were used: 50 kVp X-ray source voltage and 350 µA current, a composite X-ray filter of 1 mm aluminum, 150 ms exposure time per projection, acquiring and averaging three projections per rotation step with 0.9° increments over a total angle of 220°, and 10 cm field of view covering the whole body producing reconstructed data sets with 50 µm isotropic voxel size. Each scan took approximately 3 minutes and is associated with a measured radiation dose of 60-80 mGy. Software provided by the manufacturer (Tsort, Nrecon, DataViewer, and CTan) was used to retrospectively gate, reconstruct, visualize, and process µCT data as described by Vande Velde et al. (8). For Hounsfield unit (HU) calibration, a phantom was scanned consisting of an air-filled 1.5 ml microcentrifuge tube inside a water-filled 50 ml tube. Based on full stack histograms of a volume of interest (VOI) containing only water or air, the mean grayscale index of water [130] was set at 0 HU and grayscale index of air [9] was set at -1000 HU. Quantification of the lung volumes and respective densities was carried out for a VOI covering the lung, manually delineated on the coronal micro-CT images, avoiding the heart and main blood vessels. The threshold used to distinguish aerated from non-aerated lung tissue volume was manually set at -190 HU and kept constant for all scans.

Cytokine and chemokine detection limits

V-PLEX Proinflammatory Panel 1 Mouse Kit

| *Analyte* | *LLOD* | *LLOQ* | *ULOQ* | *Dynamic Range* | *Unit* |
| --- | --- | --- | --- | --- | --- |
| IFN-γ | 0.04 | 0.39 | 570 | 0.04 - 570 | pg/mL |
| IL-1β | 0.11 | 0.72 | 1,030 | 0.11 - 1,030 | pg/mL |
| IL-2 | 0.22 | 1.03 | 1,570 | 0.22 - 1,570 | pg/mL |
| IL-4 | 0.11 | 0.818 | 1,060 | 0.11 - 1,060 | pg/mL |
| IL-5 | 0.06 | 0.302 | 590 | 0.06 - 590 | pg/mL |
| IL-6 | 0.61 | 7.61 | 3,140 | 0.61 - 3,140 | pg/mL |
| IL-10 | 0.94 | 7.26 | 2,030 | 0.94 - 2,030 | pg/mL |
| IL-12p70 | 9.95 | 179 | 20,600 | 9.95 - 20,600 | pg/mL |
| KC/GRO | 0.24 | 3.29 | 1,230 | 0.24 - 1,230 | pg/mL |
| TNF-α | 0.13 | 0.98 | 403 | 0.13 - 403 | pg/mL |

V-PLEX Cytokine Panel 1 Mouse Kit

| *Analyte* | *LLOD* | *LLOQ* | *ULOQ* | *Dynamic Range* | *Unit* |
| --- | --- | --- | --- | --- | --- |
| IL-9 | 3.84 | 21.9 | 2,600 | 3.84 - 2,600 | pg/mL |
| IL-15 | 16 | 43.2 | 26,000 | 16.0 - 26,000 | pg/mL |
| IL-17A/F | 0.231 | 1.39 | 1,620 | 0.231 - 1,620 | pg/mL |
| IL-27p28/IL-30 | 1.39 | 5.91 | 6,500 | 1.39 - 6,500 | pg/mL |
| IL-33 | 0.364 | 1.85 | 1,950 | 0.364 - 1,950 | pg/mL |
| IP-10 | 0.328 | 2.15 | 650 | 0.328 - 650 | pg/mL |
| MCP-1 | 0.672 | 4.42 | 325 | 0.672 - 325 | pg/mL |
| MIP-1α | 0.081 | 0.38 | 390 | 0.081 - 390 | pg/mL |
| MIP-2 | 0.053 | 0.58 | 423 | 0.053 - 423 | pg/mL |

1. Xu H, Dinsdale D, Nemery B, Hoet PH. Role of residual additives in the cytotoxicity and cytokine release caused by polyvinyl chloride particles in pulmonary cell cultures. Toxicol Sci. 2003;72(1):92-102.

2. Intergewestelijke Cel voor het Leefmilieu (IRCEL) — Nederlands. [Available from: <https://www.irceline.be/nl>.

3. Ambient (outdoor) air pollution [Available from: <https://www.who.int/news-room/fact-sheets/detail/ambient-(outdoor)-air-quality-and-health>.

4. Mayeux JM, Kono DH, Pollard KM. Development of experimental silicosis in inbred and outbred mice depends on instillation volume. Sci Rep. 2019;9(1):14190.

5. Rajasinghe LD, Li QZ, Zhu C, Yan M, Chauhan PS, Wierenga KA, et al. Omega-3 fatty acid intake suppresses induction of diverse autoantibody repertoire by crystalline silica in lupus-prone mice. Autoimmunity. 2020;53(7):415-33.

6. Parks CG, Conrad K, Cooper GS. Occupational exposure to crystalline silica and autoimmune disease. Environ Health Perspect. 1999;107 Suppl 5(Suppl 5):793-802.

7. Parks CG, de Souza Espindola Santos A, Barbhaiya M, Costenbader KH. Understanding the role of environmental factors in the development of systemic lupus erythematosus. Best Pract Res Clin Rheumatol. 2017;31(3):306-20.

8. Vande Velde G, Poelmans J, De Langhe E, Hillen A, Vanoirbeek J, Himmelreich U, et al. Longitudinal micro-CT provides biomarkers of lung disease that can be used to assess the effect of therapy in preclinical mouse models, and reveal compensatory changes in lung volume. Dis Model Mech. 2016;9(1):91-8.
